# Structure-function studies of a nucleoplasmin isoform from *Plasmodium falciparum*

**DOI:** 10.1016/j.jbc.2025.108379

**Published:** 2025-03-04

**Authors:** Ketul Saharan, Somanath Baral, Surajit Gandhi, Ajit Kumar Singh, Sourav Ghosh, Rahul Das, Viswanathan Arun Nagaraj, Dileep Vasudevan

**Affiliations:** 1 Structural Biology Laboratory, BRIC-Institute of Life Sciences (BRIC-ILS), Bhubaneswar, India; 2 Regional Centre for Biotechnology, Faridabad, India; 3 Malaria Parasite Biology Laboratory, BRIC-Institute of Life Sciences (BRIC-ILS), Bhubaneswar, India; 4 Structural Biology Laboratory, BRIC-Rajiv Gandhi Centre for Biotechnology (BRIC-RGCB), Thiruvananthapuram, India

**Keywords:** crystal structure, histone chaperone, nucleoplasmin, nucleosome, plasmodium

## Abstract

An organized regulation of gene expression and DNA replication is vital for the progression of the complex life cycle of *Plasmodium falciparum* (*Pf*), involving multiple hosts and various stages. These attributes rely on the dynamic architecture of chromatin governed by several factors, including histone chaperones. Nucleoplasmin class of histone chaperones perform histone chaperoning function and participate in various developmental processes in eukaryotes. Here, our crystal structure confirmed that *Pf* indeed possesses a nucleoplasmin isoform (*Pf*NPM), and the N-terminal core domain (NTD) adopts the characteristic pentameric doughnut conformation. Furthermore, *Pf*NPM exists as a pentamer in solution, and the N-terminal core domain exhibits thermal and chemical stability. *Pf*NPM interacts individually with assembled H2A/H2B and H3/H4 with an equimolar stoichiometry, wherein the acidic tracts of *Pf*NPM were found to be necessary for these interactions. Further, H3/H4 displays a higher binding affinity for *Pf*NPM than H2A/H2B, potentially due to stronger electrostatic interactions. The interaction studies also suggested that H2A/H2B and H3/H4 might share the same binding site on the *Pf*NPM distal face, wherein H3/H4 could substitute H2A/H2B due to a higher binding affinity. Intriguingly, *Pf*NPM neither demonstrated direct interaction with the nucleosome core particles nor displayed nucleosome assembly function, suggesting it may not be directly associated with histone deposition on the parasite genomic DNA. Furthermore, our immunofluorescence results suggested that *Pf*NPM predominantly localizes in the nucleus and exhibits expression only in the early blood stages, such as ring and trophozoite. Altogether, we provide the first report on the structural and functional characterization of *Pf*NPM.

*Plasmodium falciparum* (*Pf*) causes the most fatal form of human malaria, resulting in over half a million deaths and over 200 million infections yearly (1). The high morbidity and death rate associated with the disease is attributed to the intricate life cycle of the parasite in the blood stages (2). The intraerythrocytic developmental cycle (IDC) of *Pf*, which encompasses the ring, trophozoite, and schizont stages, is responsible for malaria pathogenesis. The IDC involves the expression of over 80% of the total genes and rapid cyclic DNA replication. Notably, transcriptional regulation is tightly controlled just-in-time manner among the ring, trophozoite, and schizont stages of the IDC (3).

The eukaryotic chromatin is a blend of static and dynamic nature that switches between condensed and decondensed states, regulating the accessibility of genomic DNA for key cellular processes involving transcription, DNA replication, and repair throughout the eukaryotic system (4, 5). Previous studies in *Pf* have shown that the chromatin accessibility seen during IDC has a synergistic relationship with transcription regulation and DNA replication, suggesting that chromatin dynamics and its regulatory factors play a key role in controlling the cellular homeostasis of the parasite (6, 7, 8). Hence, to gain an in-depth understanding of the fundamental biological processes of the malaria parasite, it is crucial to examine the nuclear factors that govern the accessibility of chromatin for the necessary DNA-dependent operations in *Pf*.

Several chromatin-associated factors—including chromatin remodelers, histone modifiers, DNA modifiers, and histone chaperones—have a role in modulating the accessibility of genomic DNA to the transcriptional and replication regulatory machinery (9). Among these, histone chaperones are a structurally diverse class of acidic proteins associated with the highly basic histones postsynthesis. The basic histones tend to form aggregates upon exposure to DNA at near-physiological salt concentrations. Histone chaperones avert such nonspecific interactions and aggregation of histones, consequently aiding their deposition onto the DNA, thereby allowing a controlled and ordered assembly of the nucleosome, the fundamental unit of chromatin (10, 11).

Among the several histone chaperones characterized thus far, nucleoplasmins are the first discovered molecular chaperones found abundant in the nuclear fraction of *Xenopus laevis* oocytes (12). Nucleoplasmin family members hold a structurally well-conserved N-terminal core domain (NTD) followed by a few acidic tracts with varying numbers of aspartate and glutamate residues. The NTD has been characterized by its tendency to organize into a stable pentamer (13). The members of the nucleoplasmin family are reported to interact with the assembled H2A/H2B and H3/H4. There is also evidence demonstrating their interaction with histone octamer and nucleosome core particles (NCPs) (14, 15). Based on sequence similarity and domain organization, nucleoplasmins have been grouped into three classes: 1) classical nucleoplasmins (that includes NPM1, NPM2, and NPM3) with an NTD and C-terminal acidic tracts, 2) FK506-binding protein (FKBP) nucleoplasmins possessing a C-terminal FKBP domain in addition to the NTD and acidic tracts and 3) HD-tuin nucleoplasmins, the plant-specific nucleoplasmins, with an NTD, acidic tracts, and a possible zinc finger motif at the C terminus (16).

Mammalian NPM1 is reported to perform key cellular processes such as maintaining genomic stability (17), nuclear stress response (18), nucleocytoplasmic transport (19, 20), and chromatin remodeling (21, 22). *X. laevis* NPM2 is associated with sperm chromatin decondensation after fertilization (23). Furthermore, NPM2 from zebrafish and mice has been shown to regulate egg development (24) and centromeric assembly (25). In mammals, NPM3 regulates ribosome biogenesis (26) and paternal chromatin remodeling (27). NPM1 and NPM2 have been reported to perform histone chaperoning functions (28, 29). *Arabidopsis thaliana* FKBP53, an FKBP nucleoplasmin, performs nucleosome assembly *in vitro* (14). Although the nucleoplasmin family of proteins has been exhaustively studied across the eukaryotes, thus far, nucleoplasmins have yet to be reported in *Pf*.

Our structure-function analyses characterized a nucleoplasmin isoform from the human malaria parasite, hereafter termed *Pf*NPM. The crystal structure revealed that the NTD of *Pf*NPM adopts a β-sandwich fold and assembles into a snuggly fitted pentamer, typical of nucleoplasmins. The C-terminal acidic tracts of the *Pf*NPM NTD were found to be essential for H2A/H2B and H3/H4 interactions. Interestingly, our results suggest that the assembled H2A/H2B and H3/H4 likely share the binding site on *Pf*NPM, and the chaperone prefers H3/H4 over the H2A/H2B. However, *Pf*NPM did not show nucleosome assembly function *in vitro*. *Pf*NPM displays differential expression in IDC, with predominant localization in the nucleus, and exhibited phosphorylation PTM *in vivo*.

## Results

### Sequence, domain, and phylogenetic analysis suggest a nucleoplasmin isoform in *Pf*

**Figure 1:**
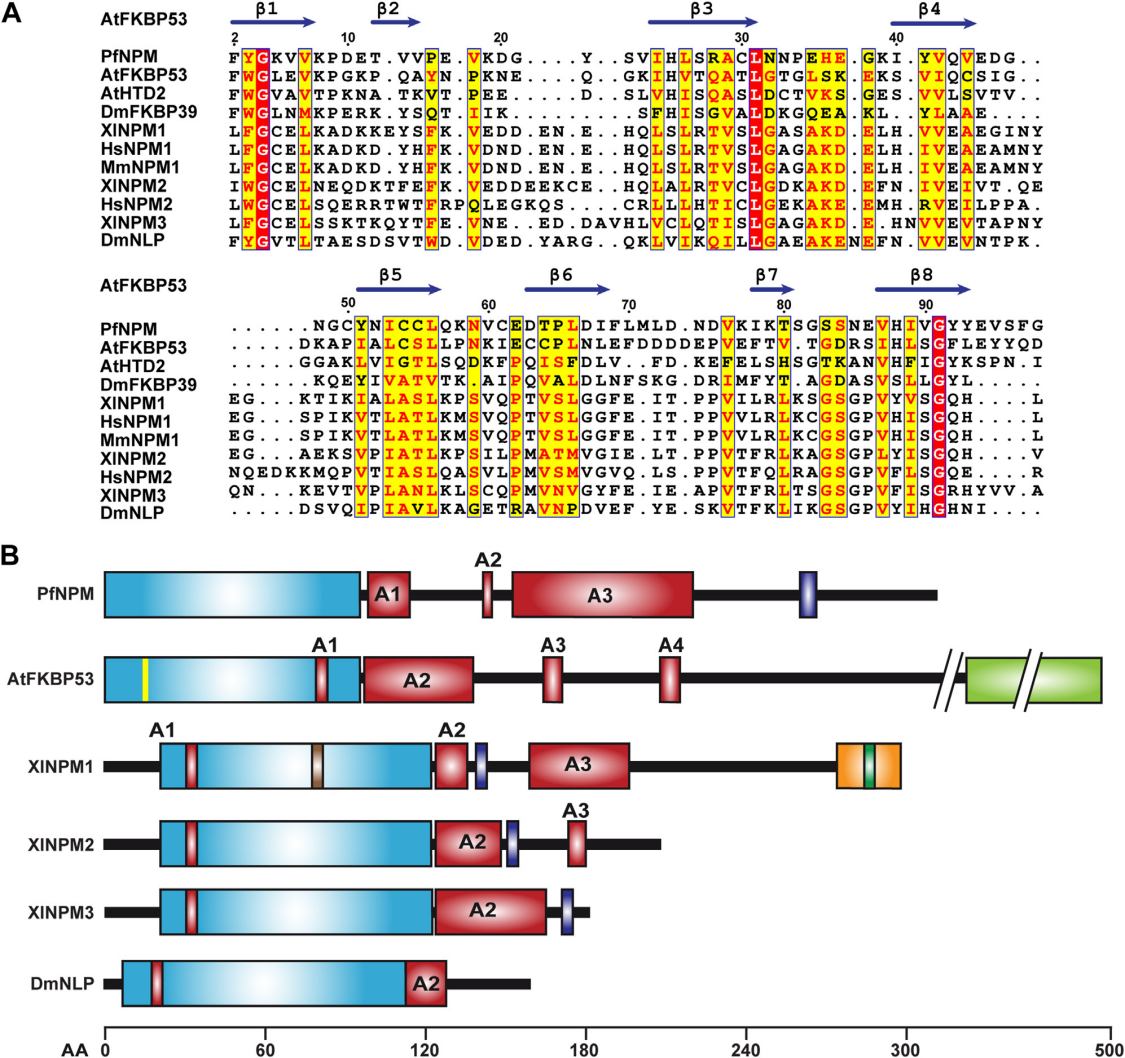
**Multiple sequence alignment and domain organization analysis of *Pf*NPM with other nucleoplasmins.***A*, the sequence comparison of *Pf*NPM NTD with the NTD of other nucleoplasmins. The UniProtKB IDs of the corresponding sequences and sequence identities of the various NTDs with *Pf*NPM (UniProtKB ID: C0H4U4) NTD are mentioned in parentheses*At*FKBP53 (Q13451; 26%), *At*HDT2 (Q56WH4; 19.2%), *Dm*FKBP39 (P54397; 21.2%), *Xl*NPM1 (P07222; 12.4%), *Hs*NPM1 (P06748; 11.9%), *Mm*NPM1 (Q5BL09; 11.5%), *Xl*NPM2 (P05221; 8.7%), *Hs*NPM2 (Q86SE8; 10.9%), *Xl*NPM3 (O42584; 16.2%), and DmNLP (Q27415; 14%). The secondary structural features of *At*FKBP53 NTD (PDB ID: 6J2Z) have been provided on top of the sequence alignment. The residue numbering is as per *Pf*NPM. *B*, the domain organization comparison of *Pf*NPM with *At*FKBP53, *Xl*NPM1, *Xl*NPM2, *Xl*NPM3, and *Dm*NLP. The NTD is shown in *blue*, the acidic tracts (A1, A2, A3, and A4) are shown in *red*, the nuclear export signal (NES) is shown in *brown*, the nuclear localization signal (NLS) is shown in *purple*, the 3_10_-helix is shown in *yellow*, the nucleic acid binding motif is shown in *orange*, the nucleolar localization signal (NoLS) is shown in *green*, and the FK506 binding domain (FKBD) is shown in *pale green*. NTD, N-terminal core domain.

A search of the PlasmoDB database using the amino acid sequence of the *A. thaliana* FKBP53 nucleoplasmin domain as a template revealed a single isoform of nucleoplasmin, *Pf*NPM (PlasmoDB ID PF3D7_0813300). Multiple sequence alignment demonstrated that *Pf*NPM NTD shares 20 to 26% sequence identity with *At*FKBP53 NTD, DmFKBP39, and AtHDT2, suggesting they possess close relatedness with FKBP and histone deacetylase tuin (HD-tuin or HDT) nucleoplasmins (Fig. 1*A*). *Pf*NPM NTD showed relatively low (below 15%) sequence identity with classical nucleoplasmins (Fig. 1*A*). However, the overall domain organization appeared quite conserved across nucleoplasmins, wherein *Pf*NPM contains a characteristic NTD, three variable-sized acidic tracts, and a C-terminal nuclear localization signal (Fig. 1*B*). Furthermore, we examined the evolutionary relatedness of the nucleoplasmin domain of *Pf*NPM with other nucleoplasmins using phylogenetic tree analysis by the maximum likelihood method. The phylogenetic analysis suggested that *Pf*NPM shares the clade having a single ancestor with FKBP nucleoplasmins and HDT nucleoplasmins, suggesting *Pf*NPM is more evolutionarily related to FKBP and HDT nucleoplasmins than classical nucleoplasmins (Fig. S1).

### *Pf*NPM NTD exhibits conserved nucleoplasmin fold

**Figure 2:**
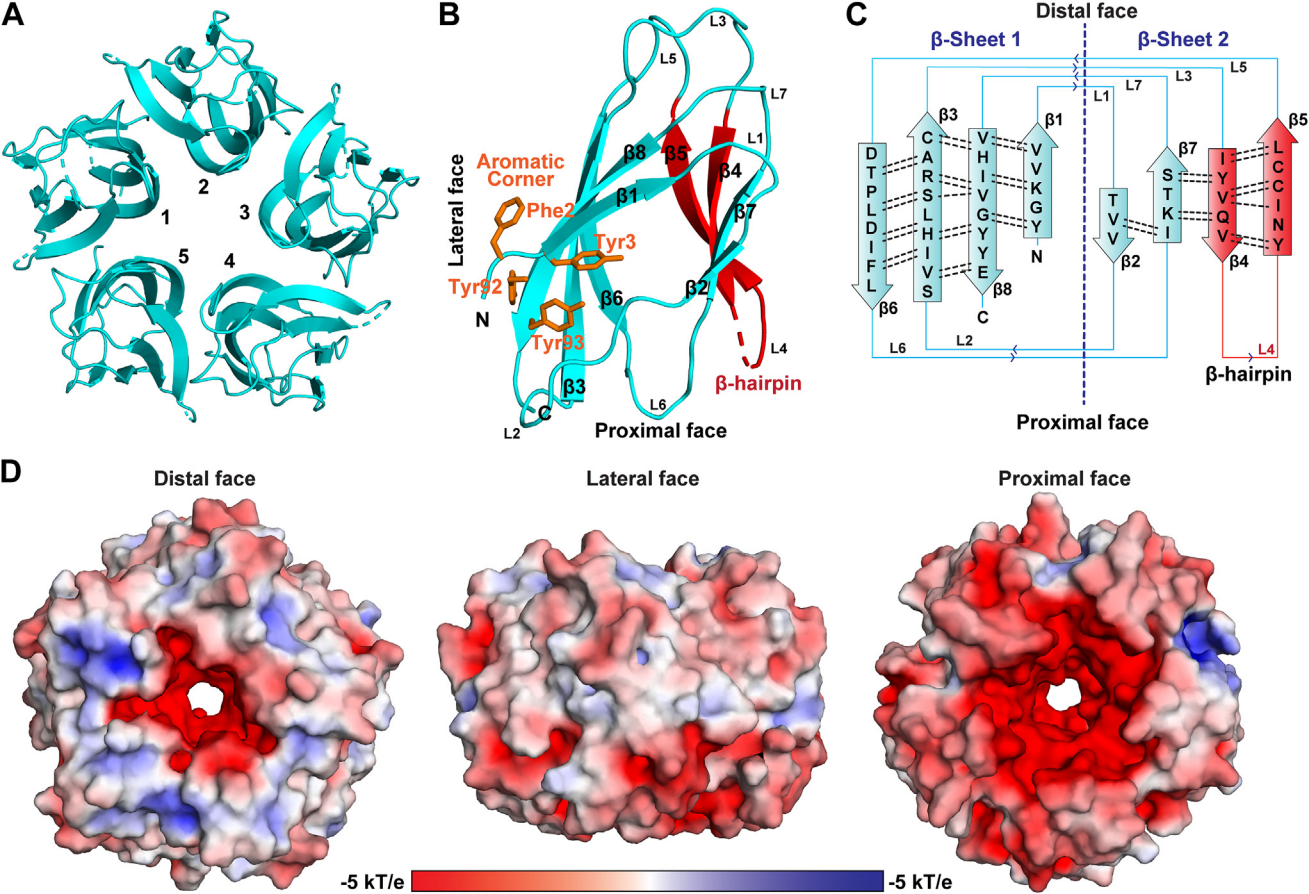
**Crystal structure of *Pf*NPM NTD.***A*, *cartoon representation* of *Pf*NPM NTD pentamer structure (*in cyan*) showing a view from the distal face. *B*, *cartoon representation* of *Pf*NPM NTD monomer (*in cyan*). The distal, proximal, and lateral faces are labeled. The β-strands and the loops are also labeled. The β-hairpin formed by β4 and β5 (*red*) and aromatic corner residues (*orange*) are also shown. *C*, the 2D topology diagram of the *Pf*NPM NTD monomer showing the β-sheet 1 and β-sheet 2 and loops connecting the β-strands. The hydrogen bond distribution in both β-sheets is shown as *dotted black lines*. *D*, the surface electrostatic properties of *Pf*NPM NTD calculated using PyMOL plugin APBS electrostatics. The proximal face (*left panel*), lateral face (*middle panel*), and distal face (*right panel*) of *Pf*NPM NTD are shown in the *surface view*. The surface view has been colored based on the electrostatic potential with *red* (−5 kT/e), *white* (0 kT/e), and *blue* (+5 kT/e). The prominent distribution of acidic residues on the proximal face provides a significant negative charge. NTD, N-terminal core domain.

The crystal structure of *Pf*NPM NTD was solved at 3.25 Å resolution and belonged to the P3_1_21 space group, wherein an asymmetric unit contained five polypeptide chains. The data collection and processing details are presented in Table 1. The *Pf*NPM NTD revealed a distinct arrangement resembling a doughnut, composed of five monomers arrayed radially along a 5-fold symmetry axis (Figs. 2*A*, S2*A*), typical of a nucleoplasmin. Each monomer features eight β-strands antiparallel to one another, yielding a β-sandwich fold arranged in two sheets: sheet-1 encompasses β1, β3, β6, and β8, while sheet-2 encompasses β2, β4, β5, and β7 (Figs. 2, *B* and *C*, S2, *B* and *C*). The β-sandwich fold is the signature of nucleoplasmin family proteins. The structural analysis revealed that both the N- and C-termini ingress and egress through the distal face of the pentamer (Fig. 2*B*). The β-strands exhibit a parallel orientation with respect to the 5-fold symmetry axis, wherein the β6 strand is situated in closest proximity while the β4-β5 hairpin is located at the farthest distance from the 5-fold axis. The β-sandwich fold is stabilized by a hydrogen bonding arrangement between neighboring antiparallel β-strands. The introduction of bulges in β8 and β5, caused by kinking and a break in β8, disrupts the typical hydrogen bonding pattern in the monomeric NTD of *Pf*NPM. The residues Phe2, Tyr3, Tyr92, and Tyr93 of all five monomers form aromatic corners toward the distal face of the structure (Fig. 2*B*). The distribution of apolar residues allows strong hydrophobic interactions between the β-sheets of a monomer and between two adjacent monomers, likely contributing to a compact and highly stable *Pf*NPM pentamer (Fig. S5, *A* and *B*).

**Table 1:** Data collection, processing, and structure refinement details of *Pf*NPM NTD

Parameters	*Pf*NPM NTD
Data collection and processing	
Beamline	ID30-A3
Detector type	Eiger X 4M
Wavelength (Å)	0.96770
Data collection temperature	100 K
Space group	P 3_1_ 2 1
a, b, c (Å)	89.35, 89.35, 113.28
α, β, γ (^o^)	90, 90, 120
Resolution (Å)	45.70 (3.25)
R_merge_	0.559 (4.466)
I/σI	10.6 (2.2)
CC (1/2) (%)	0.999 (0.778)
Total no. of reflections	8692 (1726)
Completeness (%)	99.8 (100)
Multiplicity	24.8 (26.1)
Refinement	
Wilson B-factor (Å^2^)	94.9
Solvent content (%)	56.90
No. of molecules in ASU	5
R_work_/R_free_ (%)	23.70/27.00
Total no. of non-H atoms	3623
No. of water molecules	0
Mean B-factor (Å^2^)	90.0
MolProbity score	2.00
Clash score	10.00
RMSDs	
Bond lengths (Å)	0.0079
Bond angles (^o^)	1.17
Ramachandran plot values (%)	
Favoured/Allowed/Outliers	92.24/7.76/00.00
Rotamer outliers	0

The positioning of negatively charged residues on the distal face of the NTD has been reported as crucial for the interaction of nucleoplasmins with H2A/H2B and H3/H4. Interestingly, despite lacking an A1 acidic tract, the *Pf*NPM NTD pentamer displays a noticeable negative charge distribution toward its distal end (Fig. 2*D*). This could be due to the presence of acidic residues on loops L2, L4, and L6 of the monomers exposed on the distal face of the pentamer. The proximal and lateral faces display a heterogeneous distribution of acidic and basic residues.

### Structural comparison with other nucleoplasmins

A comparison of *Pf*NPM NTD with other nucleoplasmins revealed that it holds a highly conserved structural organization, similar to other nucleoplasmins, with the RMSD values ranging between 0.80 Å and 1.85 Å when the monomers were structurally aligned (Fig. S3). Moreover, the structural alignment, taking into account both amino acid sequences and secondary structural features, demonstrated that the PfNPM pentamer exhibited significant conservation with other nucleoplasmin pentamers (Fig. S4). Although the *Pf*NPM NTD monomers possess the conserved nucleoplasmin fold, they show significant structural differences from other nucleoplasmins. The A1 acidic tract of the NTD is crucial for the histone interaction of nucleoplasmins. In loop L6, *At*FKBP53 has an A1 acidic tract (EEDEE), whereas *Xl*NPM1 has an A1 acidic tract in loop L2. In contrast, the *Pf*NPM NTD lacks a continuous acidic tract and instead has acidic residues discontinuously distributed in both the L2 and L6 loops. The presence of the GSGP and AKDE motifs on the distal face of *Xl*NPM1 has been demonstrated to cause decamerization. *Pf*NPM lacks the AKDE and GSGP motifs (Fig. S5, *G* and *H*) and exists only as a pentamer. This has been confirmed using analytical ultracentrifugation (AUC) and small angle X-ray scattering (SAXS). The 3_10_-helix of *At*FKBP53 NTD at L2 is known to stabilize the loops; however, such a 3_10_-helix is absent in the *Pf*NPM NTD crystal structure (Fig. S5*F*). The β-hairpin organization and the length of loop L4 differ significantly among nucleoplasmins. The length of the β4 and β5 strands in the β-hairpin is extended, and it protrudes away from the 5-fold symmetry axis in *Hs*NPM1, *Xl*NPM1, and *Xl*NPM2 nucleoplasmin NTDs. In the case of *Pf*NPM NTD, the β-hairpin is relatively shorter and goes parallel to the 5-fold symmetry axis (Fig. S5, *C*–*E*).

### *Pf*NPM exists as a pentamer in solution

**Figure 3:**
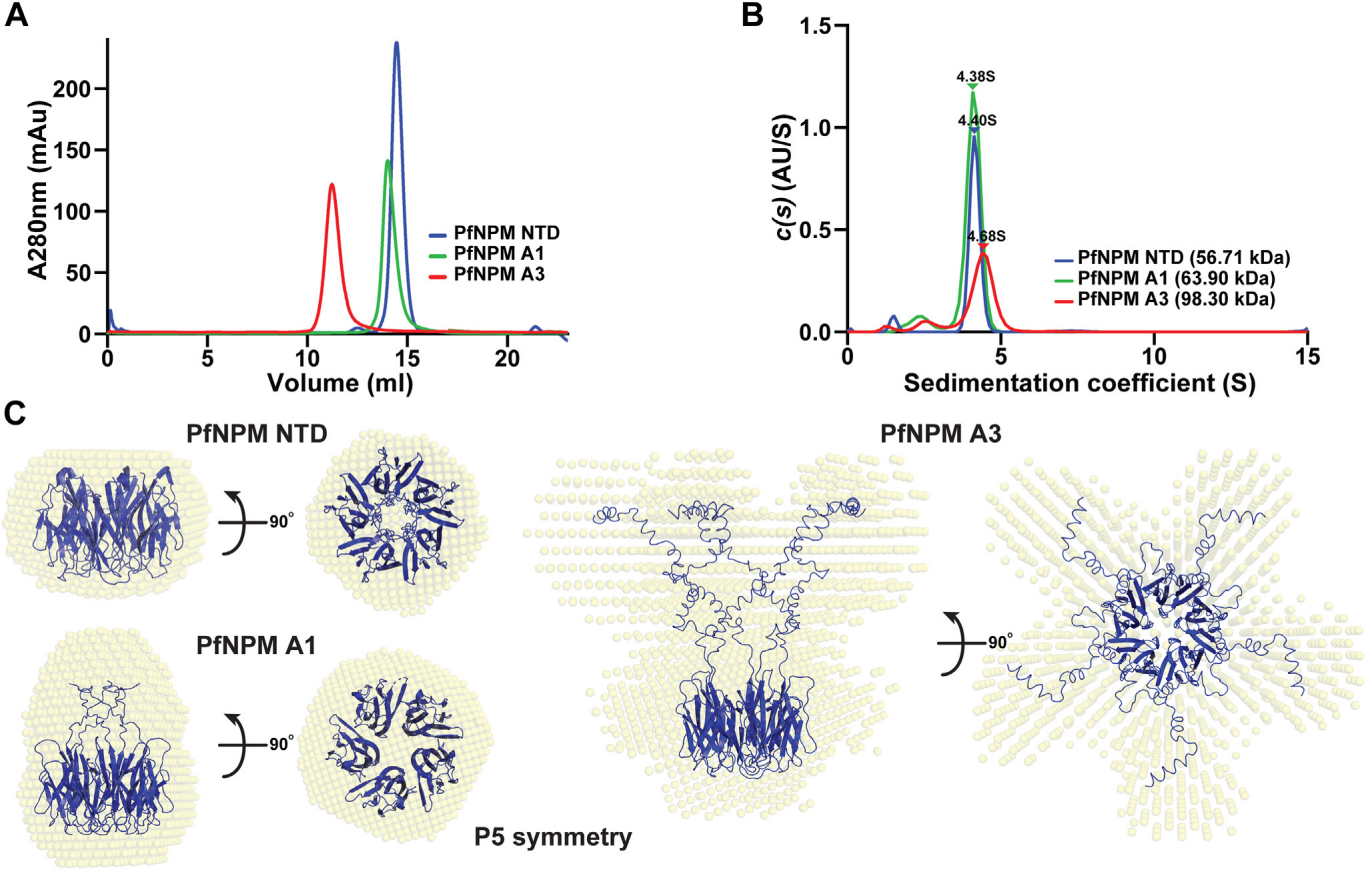
**Oligomeric state analysis of *Pf*NPM.***A*, analytical-SEC profile of *Pf*NPM NTD, *Pf*NPM A1, and *Pf*NPM A3. The results implied that all three proteins are homogenous and possess a single oligomeric state. *B*, SV-AUC continuous distribution profile of *Pf*NPM NTD, *Pf*NPM A1, and *Pf*NPM A3. The sedimentation coefficient (S, 20w) and the corresponding molecular weights obtained confirmed that *Pf*NPM NTD, *Pf*NPM A1, and *Pf*NPM A3 are indeed pentameric in nature. *C*, the low-resolution SAXS bead models (*in yellow*) of *Pf*NPM NTD, *Pf*NPM A1, and *Pf*NPM A3 were generated from DAMMIN using P5 symmetry fitted with their respective pentameric structure models (*in blue*). AUC, analytical ultracentrifugation; NTD, N-terminal core domain; SAXS, small angle X-ray scattering; SEC, size-exclusion chromatography; SV, sedimentation velocity.

A characteristic feature of the nucleoplasmin class of proteins is the existence of their pentameric oligomers in solution (16, 30, 31). To evaluate the oligomeric state of the *Pf*NPM, a set of experiments involving size-exclusion chromatography (SEC), sedimentation velocity AUC (SV-AUC), SAXS, and cryo-EM were conducted. The oligomeric state of *Pf*NPM NTD was investigated in relation to the presence of C-terminal flexible acidic tracts. To accomplish this, two variants of *Pf*NPM NTD were purified: *Pf*NPM A1 (residues 1–110), which contains up to A1 acidic tract, and *Pf*NPM A3 (residues 1–170), which contains up to A3 acidic tract. *Pf*NPM NTD, *Pf*NPM A1, and *Pf*NPM A3 analytical SEC profiles yielded uniform single peaks, indicating that the individual proteins display single oligomeric forms (Fig. 3*A*). The SV-AUC experiment yielded sedimentation coefficients of 4.38 S, 4.40 S, and 4.68 S for *Pf*NPM NTD, *Pf*NPM A1, and *Pf*NPM A3, respectively, corresponding to molecular weights of 56.71 kDa, 63.90 kDa, and 98.30 kDa (Figs. 3*B*; S6, *A*–*C*; Table S1). The SV-AUC estimated molecular weight corresponded to the pentameric state of the *Pf*NPM variants.

Subsequently, the SAXS technique was employed to investigate the low-resolution envelope architectures of the *Pf*NPM constructs. The Guinier plot showed that the samples are homogeneous and aggregation-free (Fig. S6*D*). An absolute Gaussian curve in the Kratky plot for *Pf*NPM NTD indicated that the NTD is entirely folded, while the progressive increase in the curve toward the high q region for *Pf*NPM A1 to A3 implies that the folded domain includes flexible acidic tracts (Fig. S6*E*). Distance distribution plots indicated a Dmax value of 6.97 nm, 8.60 nm, and 14.54 nm for *Pf*NPM NTD, *Pf*NPM A1, and *Pf*NPM A3, respectively (Fig. S6*F*). The calculated molecular weight of *Pf*NPM NTD, derived from the Porod volume (Vp) obtained from SAXS, was in good agreement with the theoretical molecular weight (Table S2). The molecular weight of *Pf*NPM A1 and *Pf*NPM A3 deviated slightly from the theoretical values, likely attributable to the high degree of flexibility of the acidic tracts. The beaded envelope derived from the SAXS data of *Pf*NPM NTD, *Pf*NPM A1, and *Pf*NPM A3 exhibited a good fit with their corresponding structural models (Fig. 3*C*). In addition, we also attempted to determine the structure of *Pf*NPM using cryo-EM. For this, PfNPM A3 was frozen on the grid, and micrographs were acquired. The image processing yielded multiple 2D class averages that likely highlight both distal and proximal views (Fig. S6*G*; Table S3). However, due to extreme flexibility, the acidic tails were unrecognizable in contrast to the clearly visible pentameric core. Nevertheless, the limited resolution and absence of lateral views imposed constraints on our ability to reconstruct a 3D map with confidence using 2D class averages. However, the 2D class averages confirmed the pentameric nature of the *Pf*NPM, thus providing additional support to the AUC and SAXS results.

### *Pf*NPM NTD displays thermal and chemical stability features

Reports suggest that nucleoplasmin pentamer demonstrates high thermal and chemical stability, the reason being the distribution of apolar and hydrophobic residues on the inner and outer layers of the core of pentamer (14, 32, 33). The analysis of the sequence and the structure of *Pf*NPM NTD substantiated the distribution of apolar residues in a similar fashion to other nucleoplasmins (Fig. S5, *A* and *B*). Analytical SEC was used to test the thermal and chemical stability attributes of *Pf*NPM NTD at increasing temperature, salt, and urea concentrations. The homogenous sharp profile of *Pf*NPM up to 60 °C demonstrated a stable pentamer and a further increase in temperature resulted in delayed elution, suggesting the pentamer might have dissociated into a lower oligomer above 60 °C (Fig. S7*A*). Additionally, the elution profile demonstrated that the pentameric form of *Pf*NPM NTD remains stable when exposed to salt concentrations of up to 2.0 M and urea concentrations of up to 2.0 M. Elevating the concentration of urea beyond 2.0 M led to the emergence of an extra peak, indicating the disintegration of pentamer into smaller oligomeric species (Fig. S7, *B* and *C*).

### Acidic tails mediate the interaction of *Pf*NPM with H2A/H2B and H3/H4

Previous research has demonstrated that the nucleoplasmin family of histone chaperones engages in interactions with H2A/H2B and H3/H4. Our structural and biophysical investigations revealed that *Pf*NPM is a member of the nucleoplasmin family proteins. Therefore, using different biochemical and biophysical techniques, the ability of *Pf*NPM to bind to assembled H2A/H2B and H3/H4 was studied. Similar to previous reports on *At*FKBP43 NTD (33) and *At*HDT2 NTD (32), our pull-down studies suggested that *Pf*NPM NTD does not interact with the assembled H2A/H2B and H3/H4. Further, we examined the role of acidic tracts of *Pf*NPM in facilitating histone interaction. The pull-down experiment indicated that *Pf*NPM A1 and *Pf*NPM A3 associate with assembled H2A/H2B and H3/H4, implying that the acidic tracts are indispensable for *Pf*NPM NTD to associate with the H2A/H2B and H3/H4 (Fig. S8, *A* and *B*).

**Figure 4:**
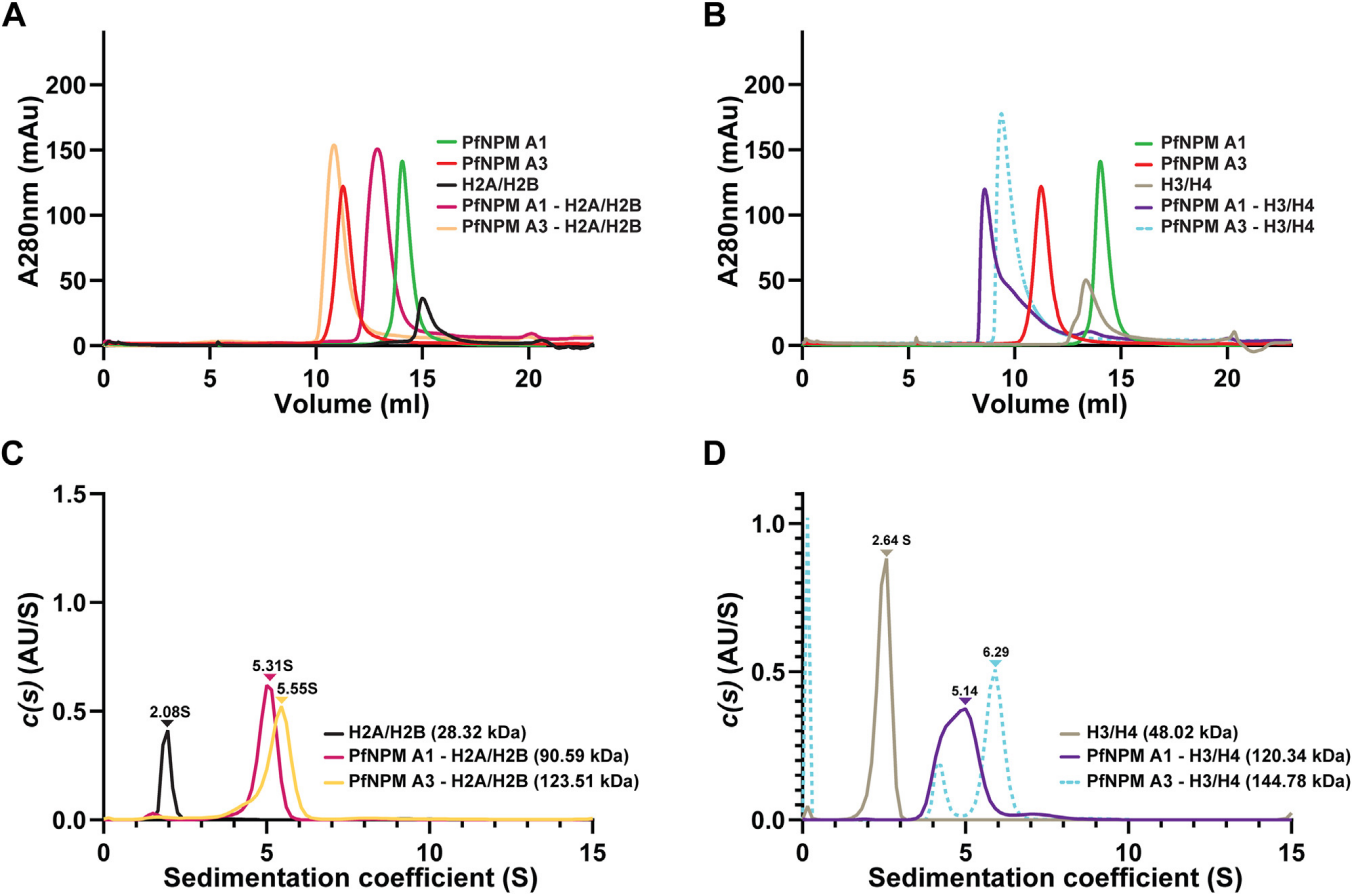
**Analysis of *Pf*NPM interactions with H2A/H2B and H3/H4 using analytical SEC and SV-AUC.***A*, analytical-SEC profile of *Pf*NPM A1 (*green*), *Pf*NPM A3 (*magenta*), assembled H2A/H2B (*black*), *Pf*NPM A1–H2A/H2B mixture (*pink*), and *Pf*NPM A3–H2A/H2B mixture (*light orange*). The early elution of *Pf*NPM A1–H2A/H2B and *Pf*NPM A3–H2A/H2B mixtures suggested the formation of their respective stable complexes. *B*, analytical-SEC profile of *Pf*NPM A1 (*green*), *Pf*NPM A3 (*magenta*), assembled H3/H4 (*light brown*), *Pf*NPM A1–H3/H4 mixture (*violet*), and *Pf*NPM A3–H3/H4 mixture (*dotted light blue*). The early elution of *Pf*NPM A1–H3/H4 and *Pf*NPM A3–H3/H4 mixtures suggested the formation of their stable complexes. *C*, SV-AUC continuous distribution profile of assembled H2A/H2B (*black*), *Pf*NPM A1–H2A/H2B (*pink*), and *Pf*NPM A3–H2A/H2B (*light orange*). The sedimentation coefficient and the corresponding molecular weights confirmed that *Pf*NPM A1 and *Pf*NPM A3 form stable complexes with H2A/H2B dimer in an equimolar stoichiometry. *D*, SV-AUC continuous distribution profile of assembled H3/H4 (*light brown*), *Pf*NPM A1–H3/H4 (*violet*), and *Pf*NPM A3–H3/H4 (*dotted light blue*). The sedimentation coefficient and the corresponding molecular weights confirmed that *Pf*NPM A1 and *Pf*NPM A3 form stable complexes with assembled H3/H4 tetramer in an equimolar stoichiometry. AUC, analytical ultracentrifugation; SEC, size-exclusion chromatography; SV, sedimentation velocity.

Analytical SEC was carried out to further characterize *Pf*NPM A1 and *Pf*NPM A3 complexes with assembled H2A/H2B and H3/H4. The *Pf*NPM A1 and *Pf*NPM A3 incubated with assembled H2A/H2B and H3/H4 individually displayed a shift in the elution volume, suggesting that *Pf*NPM A1 and *Pf*NPM A3 firmly associate with the H2A/H2B and H3/H4 (Fig. 4, *A* and *B*). Further, SV-AUC experiment revealed molecular weights of 90.59 kDa, 123.51 kDa, 120.34 kDa, and 144.78 kDa for *Pf*NPM A1–H2A/H2B, *Pf*NPM A3–H2A/H2B, *Pf*NPM A1–H3/H4, and *Pf*NPM A3–H3/H4, respectively, implying that *Pf*NPM A1 and *Pf*NPM A3 exhibit 1:1 binding stoichiometry for complex formation with both H2A/H2B dimer and H3/H4 tetramer (Figs. 4, *C* and *D*; S8, *C*–*H*; Table S4).

**Figure 5:**
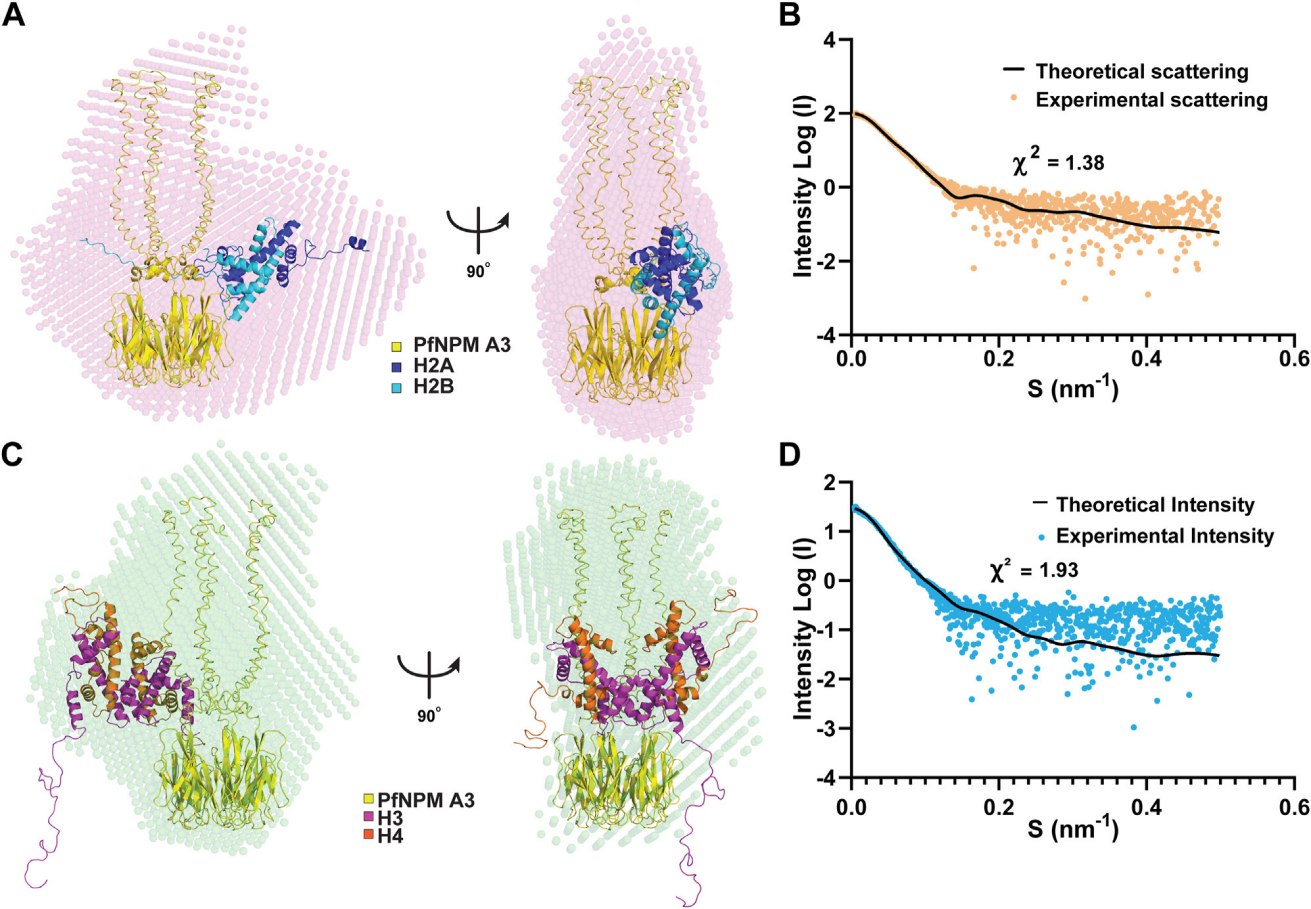
**Small-angle X-ray scattering analysis of *Pf*NPM A3–histone complexes.***A*, the low-resolution SAXS bead model of *Pf*NPM A3–H2A/H2B complex generated using DAMMIN with P5 symmetry fit to best rigid body model of *Pf*NPM A3–H2A/H2B complex calculated from FoXSDock server. *B*, the scattering curve from the experimental SAXS profile of *Pf*NPM A3–H2A/H2B complex (*orange*) overlaid with the theoretical scattering curve from the rigid body model (*black line*) calculated from FoXSDock, with the corresponding χ^2^ value, is presented. *C*, the low-resolution SAXS bead model of *Pf*NPM A3–H3/H4 complex generated using DAMMIN with P5 symmetry fit to the best rigid body model of *Pf*NPM A3–H3/H4 complex calculated from FoXSDock server. *D*, the scattering curve from the experimental SAXS profile of *Pf*NPM A3–H3/H4 complex (*light blue*) overlaid with the theoretical scattering curve from the rigid body model (*black line*) calculated from FoXSDock, with the corresponding χ^2^ value, is presented.

Further, the overall low-resolution structure of the *Pf*NPM A3 in complex with assembled H2A/H2B and H3/H4 was examined using SAXS. The sample homogeneity and the folded nature were confirmed using the Guinier and Kratky plots, respectively (Fig. S8, *I* and *J*). *Pf*NPM A3 complex with assembled H2A/H2B and H3/H4 individually yielded Dmax values of 13.9 nm and 15.8 nm, respectively (Fig. S8*K*). The averaged DAMAVER bead model generated using the DAMMIN exhibited a good agreement with the structure model for *Pf*NPM A3–H2A/H2B and *Pf*NPM A3–H3/H4 complexes, as evidenced by the goodness of fit (χ2) values of 1.38 and 1.93, respectively (Fig. 5, *A*–*D*; Table S5). Furthermore, the molecular weight evaluated for *Pf*NPM A3–H2A/H2B and *Pf*NPM A3–H3/H4 complexes from SAXS supported the binding stoichiometry observed in AUC analysis.

### *Pf*NPM shows a higher binding affinity for H3/H4 than H2A/H2B

**Figure 6:**
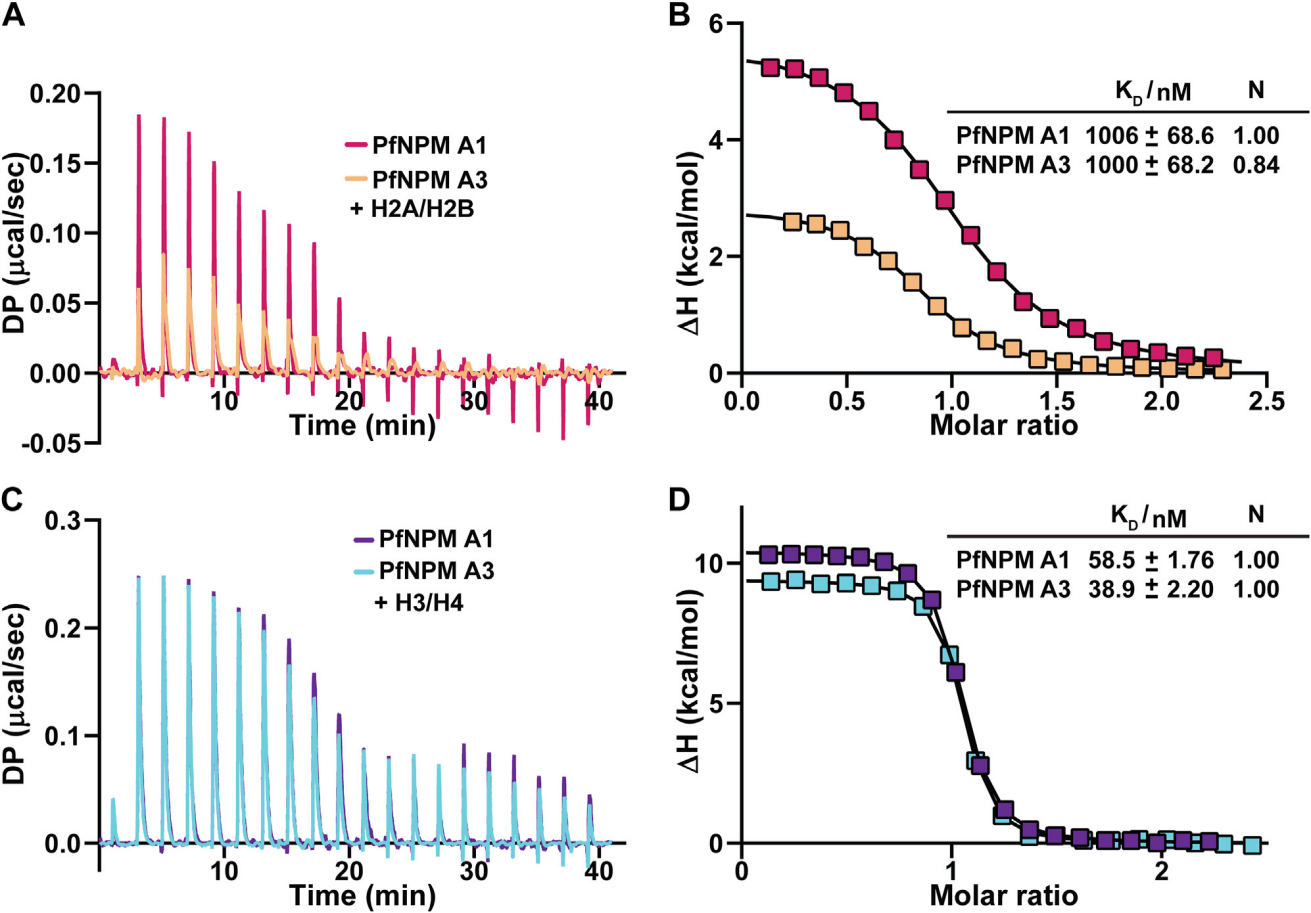
**Isothermal titration calorimetry analyses for *Pf*NPM with assembled H2A/H2B and H3/H4.** The binding of *Pf*NPM A1 and *Pf*NPM A3 with assembled H2A/H2B and H3/H4 was examined using isothermal titration calorimetry. *A*, the titration of H2A/H2B into *Pf*NPM A1 and *Pf*NPM A3 is shown as a raw heat change plot. *B*, the titration of H2A/H2B into *Pf*NPM A1 and *Pf*NPM A3 is shown as an integrated heat plot. The binding affinities and number of binding sites “N” are shown in parenthesis. *Pf*NPM A1 and *Pf*NPM A3 showed comparable binding affinities for H2A/H2B. *C*, the titration of H3/H4 into *Pf*NPM A1 and *Pf*NPM A3 is shown as a raw heat change plot. *D*, the titration of H3/H4 into *Pf*NPM A1 and *Pf*NPM A3 is shown as an integrated heat plot. The binding affinities and number of binding sites “N” are shown in parenthesis. *Pf*NPM A1 and *Pf*NPM A3 showed comparable binding affinities for H3/H4; however, 20-fold excess than that for H2A/H2B.

We next used isothermal titration calorimetry (ITC) and pull-down assays to assess the binding affinity and binding preference of *Pf*NPM for assembled H2A/H2B and H3/H4. Utilizing an ITC experiment, the binding affinity and thermodynamics of *Pf*NPM for its histone interaction were analyzed. The titration of *Pf*NPM A1 and *Pf*NPM A3 into assembled H2A/H2B individually resulted in an endothermic reaction with a K_d_ value of 1006 ± 68.6 nM and 1000 ± 68.2 nM, respectively (Fig. 6, *A* and *B*). The titration of *Pf*NPM A1 and *Pf*NPM A3 into assembled H3/H4 individually resulted in an endothermic reaction with the K_d_ value of 58.5 ± 1.76 nM and 38.9 ± 2.20 nM, respectively (Fig. 6, *C* and *D*). The titrations resulted in unfavourable ΔH (+ve) and favourable ΔS (−ve), suggesting the interaction of both H2A/H2B and H3/H4 with *Pf*NPM is driven by favourable entropy (Fig. S9, *A*–*D*). The binding stoichiometry of 1:1 for both *Pf*NPM with assembled H2A/H2B and H3/H4 obtained from ITC, corroborated well with the AUC results.

**Figure 7:**
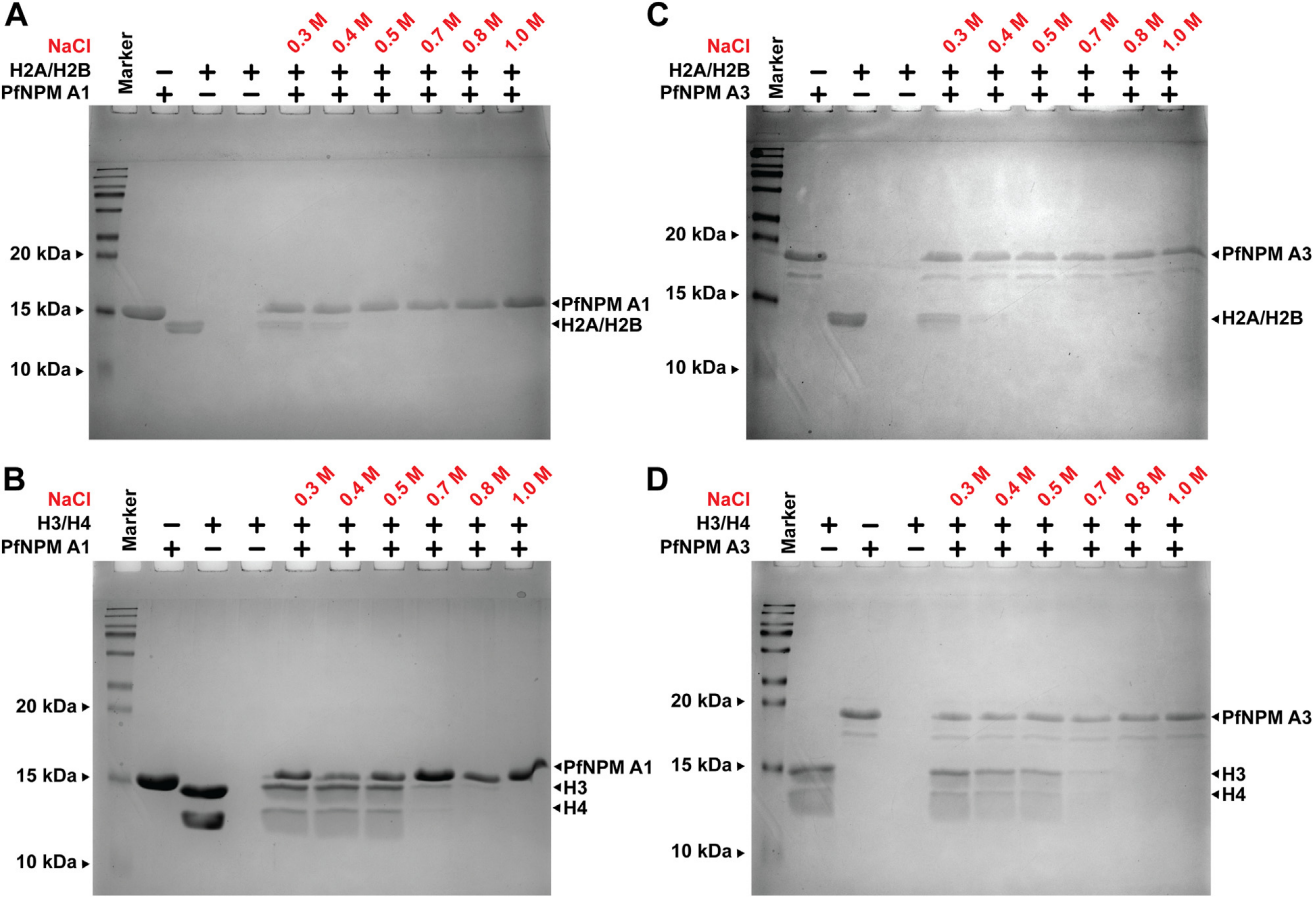
**Salt gradient pull-down assay for stability analysis of *Pf*NPM complexes with assembled H2A/H2B and H3/H4.** The elution fractions recovered from the pull-down assay of (*A*) *Pf*NPM A1–H2A/H2B complex and (*B*) *Pf*NPM A3–H2A/H2B complex were separately subjected to an 18% SDS-PAGE and stained with Coomassie brilliant *blue*. H2A/H2B in complex with *Pf*NPM A1 and *Pf*NPM A3 was stable up to 400 mM NaCl. The elution fractions recovered from the pull-down assay of (*C*) *Pf*NPM A1–H3/H4 complex and (*D*) *Pf*NPM A3–H3/H4 complex were separately subjected to an 18% SDS-PAGE and stained with Coomassie brilliant *blue*. H3/H4 in complex with *Pf*NPM A1 and *Pf*NPM A3 showed stability up to 600 mM NaCl.

The ITC results suggested that *Pf*NPM exhibits approximately 20-fold higher binding affinity for assembled H3/H4 than assembled H2A/H2B. We investigated the interactions providing enhanced stability to the *Pf*NPM–H3/H4 complex over the H2A/H2B complex. The salt gradient pull-down experiment results indicated that the *Pf*NPM–H2A/H2B complex was stable only up to 400 mM NaCl, whereas the *Pf*NPM–H3/H4 complex was stable up to 600 mM NaCl, suggesting that probably increased electrostatic interactions are responsible for the increased stability of *Pf*NPM–H3/H4 complex over H2A/H2B complex (Fig. 7, *A*–*D*).

Further, to examine the preference of histone binding by *Pf*NPM, a competitive pull-down assay was performed. The competitive pull-down assay results indicated that when incubated simultaneously, H3/H4 tetramer can substitute H2A/H2B dimer for both *Pf*NPM A1 and *Pf*NPM A3 (Fig. S10, *A*–*F*). The results suggest that the H2A/H2B dimer and H3/H4 tetramer might share the same binding site on the proximal acidic tracts of *Pf*NPM, and the H3/H4 has a higher affinity for the association.

### *Pf*NPM lacks nucleosome interaction and nucleosome assembly function *in vitro*

**Figure 8:**
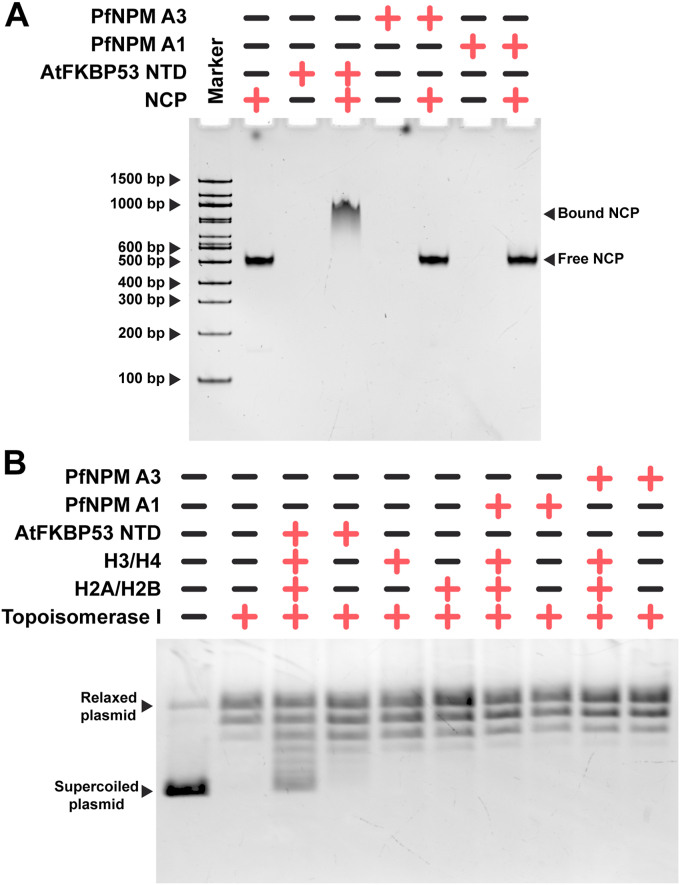
**Analysis of nucleosome interaction and nucleosome assembly properties of *Pf*NPM.***A*, electrophoretic mobility shift assay (EMSA) of *Pf*NPM A1 and *Pf*NPM A3 with NCP in a 10:1 ratio on a 6% Native-PAGE gel stained with SYBR Safe DNA gel stain. *Pf*NPM did not show interaction with NCP. *At*FKBP53 NTD with NCP in a 2:1 ratio was taken as a positive control for the assay. *B*, analysis of nucleosome assembly function of *Pf*NPM using plasmid supercoiling assay. *Pf*NPM A1 and *Pf*NPM A3 were independently incubated with the relaxed plasmid, with and without assembled H2A/H2B and H3/H4 and subjected to 1% agarose gel electrophoresis and the gel stained with SYBR Safe DNA gel stain. *Pf*NPM did not show histone deposition on the DNA. *At*FKBP53 NTD was used as a positive control in the assay. NCP, nucleosome core particle; NTD, N-terminal core domain.

The nucleoplasmin protein family is recognized for its role in both *in vivo* and *in vitro* nucleosome assembly (14, 21). Our electrophoretic mobility shift assay results inferred that *Pf*NPM does not show any apparent interaction with the reconstituted NCPs, unlike *At*FKBP53 (Fig. 8*A*). Moreover, the nucleosome assembly function of *Pf*NPM was explored using a plasmid supercoiling assay. *Pf*NPM demonstrated no supercoiling activity, suggesting that it does not aid the deposition of H2A/H2B and H3/H4 onto the DNA by itself (Fig. 8*B*).

### *Pf*NPM shows nuclear localization during intraerythrocytic development

Anti-*Pf*NPM polyclonal antibodies were generated using the purified NTD of *Pf*NPM recombinantly expressed in *Escherichia coli*. Western blot analysis was carried out to assess the specificity of the anti-*Pf*NPM antibody. Notably, compared to its original molecular weight (35 kDa), the native *Pf*NPM from parasite lysate demonstrated migration at a higher molecular weight (∼between 70 and 100 kDa) (Fig. S11*A*). Nucleoplasmins are reported to undergo phosphorylation posttranslational modification. Thus, we investigated the phosphorylation mark on *Pf*NPM protein using an anti-pan phospho antibody. Intriguingly, the detected band for the anti-pan phospho antibody exactly overlapped with the band detected for the anti-*Pf*NPM antibody, suggesting that *Pf*NPM undergoes phosphorylation within the parasite (Fig. S11*B*). The delayed migration of endogenous *Pf*NPM is likely attributed to its high negative charge (pI 4.11) and multiple phosphorylation events on the acidic tail (34, 35).

**Figure 9:**
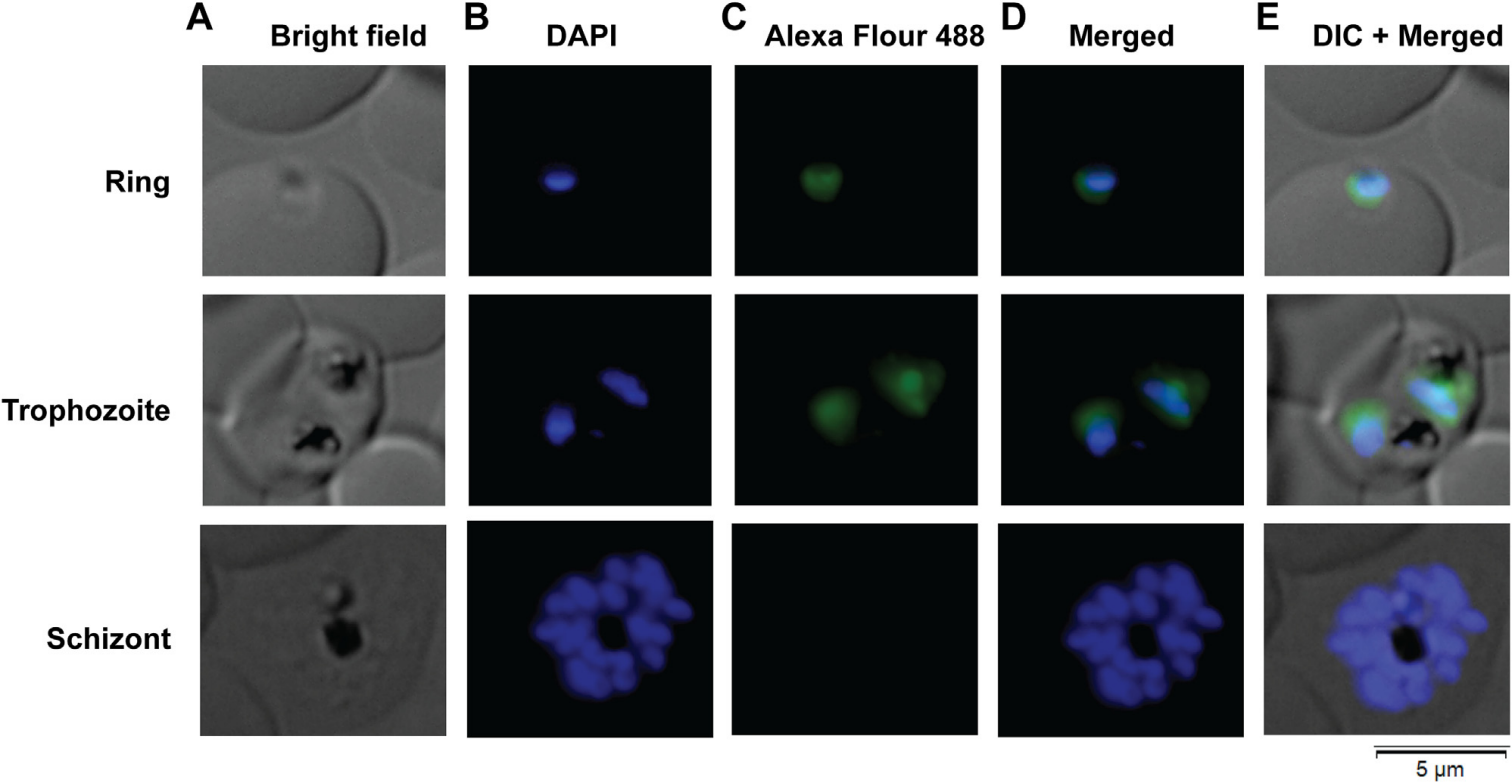
**Localization of *Pf*NPM in the blood stages of *Pf*.***Pf*NPM immunofluorescence microscopy in blood stage rings, trophozoites, and schizonts. *A*, bright field, (*B*) DAPI stained nucleus, (*C*) Alexa Flour 488–stained anti-*Pf*NPM, (*D*) DAPI overlay Alexa Flour 488 signal, (*E*) DAPI and Alexa Flour 488 signal combined with differential interference contrast (DIC). DAPI was used to stain the parasite nucleus. Alexa Fluor 488 was used to stain anti-*Pf*NPM antibodies. *Pf*NPM was found mostly in the nucleus of rings and trophozoites. Schizonts, on the other hand, lacked *Pf*NPM expression in the nucleus and cytoplasm. DAPI, 4',6-diamidino-2-phenylindole; Pf, Plasmodium falciparum.

The sequence analysis indicated the presence of a monopartite nuclear localization signal "_227_**KKK**GGD**KKKNKR**S_240_" at the C terminus of *Pf*NPM (Fig. 1*B*). In order to investigate the subcellular distribution of *Pf*NPM, an immunofluorescence (IF) assay was performed. The findings indicated predominant localization of *Pf*NPM within the nucleus of ring and trophozoite, the early-stage blood parasites. A weak signal was also observed in the cytoplasm. Nonetheless, the parasites in the schizont stage did not show any signal for *Pf*NPM (Fig. 9).

## Discussion

*Malaria* is a major public health problem causing high mortality due to the substantial acquisition of resistance against a wide range of antimalaria drugs. To advance the development of effective and novel intervention approaches, a significant improvement in our understanding of the fundamental biological processes of *Pf* is necessary. The tight gene expression and DNA replication controlled through dynamic chromatin pave the way for the effective progression of the complex life cycle of *Pf*. Histone chaperones, including nucleoplasmins, play a crucial role in regulating the dynamic structure of chromatin by assisting in the assembly and disassembly of nucleosomes. In this context, we have performed a detailed study to address the structural and functional aspects of an uncharacterized *Pf* nucleoplasmin isoform. A previous high-throughput PiggyBac mutational screening suggested that this uncharacterized protein could be indispensable for the survival of the blood-stage parasite (36).

The C-terminal acidic tracts of the nucleoplasmins are the target for phosphorylation, and these posttranslational modifications have been reported to enhance the histone binding and nucleosome assembly function (37). Proteomic analysis at IDC has shown five serine phosphorylation and three threonine phosphorylation sites (PlasmoDB; (38, 39)). The acidic nature and phosphorylation of the protein are known to retard the migration of the protein in SDS-PAGE, explaining the migration of *Pf*NPM corresponding to higher molecular weight (34, 35, 40, 41). In fact, the *Xenopus* oocyte-isolated nucleoplasmins are heavily phosphorylated and display retarded migration compared to the dephosphorylated nucleoplasmins (42, 43). A comprehensive cell-based study is required to fully elucidate the role of phosphorylation in modulating the interaction of *Pf*NPM with histone oligomers within the context of the *Pf* cellular environment.

Besides nuclear processes, histone chaperones are also linked with safeguarding histones against nonspecific interaction in the cytoplasm, followed by their transport into the nucleus (44). Our IF studies showed the predominant localization of *Pf*NPM in the nucleus with a minor signal detected from the cytosol, indicating their association with the nuclear import of histones. This finding aligns with the previous report on NASP, ASF1, and NAP1, associated with the nuclear shuttling of histones from the cytosol (44). More intriguingly, the IF and Western blotting experiments revealed that the expression of *Pf*NPM was limited to the early blood stages of the parasite, including the rings and trophozoites. Our results resonate well with previous transcriptomic and proteomic investigations, demonstrating substantially higher levels of *Pf*NPM mRNA and proteins in the rings and trophozoites than the schizonts (PlasmoDB; (45, 46, 47)).

Our structural investigation studies confirmed that a *Pf*NPM NTD monomer folds into a classical β-sandwich fold and assembles into a snuggly fit pentamer. Though they exhibit low sequence identity, structural comparison suggested no major structural differences compared to nucleoplasmins from humans, arthropods, and plants (30, 31, 32, 48, 49). However, unlike previously reported for *Xl*NPM1 and *Xl*NPM2, the crystal structure of *Pf*NPM revealed a pentameric organization and no decamers, potentially owing to the absence of GSGP and AKDE loops toward the distal face of *Pf*NPM (48, 50). Furthermore, our AUC, SAXS, and cryo-EM studies substantiated that *Pf*NPM exists as a pentamer in solution, consistent with the previous reports on certain other nucleoplasmins (14, 16, 32, 33, 51). The pentameric state of nucleoplasmins likely enables them to associate with multiple histone ligands (15, 33), in contrast to certain other histone chaperones like CAF, ASF, and NAP, which exhibit selectivity toward either H2A/H2B or H3/H4 (52, 53). The *Pf*NPM NTD pentamer exhibits notable thermal and chemical stability, potentially due to uniformly dispersed apolar residues among the monomers forming the nucleoplasmin pentamer parallel to previously reported nucleoplasmins (14, 32, 33, 48, 49). However, the significance of the extensive thermal and chemical stability attributes on the physiology of the parasite warrants investigation. The extensive thermal stability of *Pf*NPM may support its functionality when the blood-stage parasites are exposed to febrile temperatures in IDC.

The interaction studies demonstrated that *Pf*NPM can individually interact with assembled H2A/H2B and H3/H4. Our findings revealed that at least the A1 acidic tract with *Pf*NPM NTD is crucial for its interaction with H2A/H2B and H3/H4. Apart from *At*FKBP53, all previously documented nucleoplasmins—including *Xl*NPM2, *At*FKBP43, and *At*HDT2—have exhibited the indispensability of their acidic tracts for histone interactions (14, 32, 33). The plausible justification for the inability to interact with H2A/H2B and H3/H4 by *Pf*NPM NTD lacking an A1 acidic tract is a relatively weak negative charge on the proximal face. A negative charge on the proximal face is typically deemed crucial in facilitating interactions of nucleoplasmins with positively charged histones (33, 54, 55).

SV-AUC and SAXS revealed 1:1 binding stoichiometry of *Pf*NPM for its interaction with H2A/H2B and H3/H4, suggesting that one *Pf*NPM pentamer binds to one H2A/H2B dimer and one H3/H4 tetramer individually. Interestingly, the binding stoichiometry of *Pf*NPM with H2A/H2B and H3/H4 compares well with FKBP and HDT nucleoplasmins, whereas, contrasting to the 1:5 binding stoichiometry observed for *Xl*NPM2, a canonical nucleoplasmin (14, 32, 33, 54, 55, 56). Furthermore, the SAXS obtained Dmax and the SV-AUC–derived friction ratio suggests that the complexes adopt a more globular and compact nature than their constituents. The SAXS-derived structure model implies that H2A/H2B and H3/H4 get possibly accommodated on the acidic tracts at the proximal face. Nevertheless, understanding the atomic details of complex formation requires high-resolution structural investigations.

The binding affinities of *Pf*NPM A1 and *Pf*NPM A3 for H2A/H2B are comparable. Likewise, the affinities of *Pf*NPM A1 and *Pf*NPM A3 for H3/H4 also exhibit a comparable affinity. ITC experiments suggested a greater binding affinity of *Pf*NPM toward H3/H4 tetramer than *At*FKBP53, *At*FKBP43, and *At*HDT2, as reported in previous studies (14, 32, 33). In comparison, *Pf*NPM exhibited a binding affinity toward H2A/H2B in the micromolar range, which is approximately 2-fold lower than the binding affinities reported for *At*FKBP53, *At*FKBP43, and *At*HDT2 (14, 32, 33). Furthermore, the phosphorylation posttranslational modifications are also vital in controlling the binding affinity for histone chaperones (37). For instance, the hyperphosphorylated NPM2 isolated from the *Xenopus* egg displays a subnanomolar binding affinity for H2A/H2B and nanomolar binding affinity for H3/H4 (57). Though the exact reason is obscure, the involvement of different extents of hydrophobic and polar interactions might be a reason for the difference in the binding affinities of H2A/H2B and H3/H4 among different nucleoplasmins.

A combination of ITC and salt gradient pull-down analysis suggested that due to a greater extent of hydrophobic and electrostatic interactions, the H3/H4 tetramer exhibits a higher binding affinity for *Pf*NPM than the H2A/H2B dimer. Though nucleoplasmins initially were reported to interact with H2A/H2B, subsequent reports confirmed that they can associate with H3/H4 as well (13, 23). Nonetheless, studies demonstrating the competitive nature of H2A/H2B and H3/H4 to nucleoplasmins are lacking. Our competitive pull-down assay indicated that though both H2A/H2B and H3/H4 share the binding site on the *Pf*NPM, H3/H4 can substitute H2A/H2B on the binding site, probably due to a higher extent of electrostatic and hydrophobic interactions. However, further research is necessary to ascertain the physiological significance of the mode of histone preference exhibited by *Pf*NPM in the parasite.

Based on their concomitance with cell cycle progression and DNA replication process, the histone chaperones are categorized into replication-dependent and replication-independent histone chaperones (58, 59). Trophozoite is the most metabolically active stage in the parasite IDC, with active transcription and protein synthesis (60), whereas the schizonts are known for active DNA replication and synthesis (61). The expression of *Pf*NPM in rings and trophozoites suggests that the *Pf*NPM function is most likely associated with transcription regulation. Our *in vitro* assembly assays revealed that *Pf*NPM does not directly perform nucleosome assembly functions, unlike a few other histone chaperones (10, 62). High-throughput yeast two-hybrid analyses revealed direct interaction of *Pf*NPM with the Spt16 subunit of the histone chaperone facilitates chromatin transcription (FACT) (PlasmoDB; (63)). FACT is known to facilitate the process of transcription by assisting in the assembly and disassembly of nucleosomes during active gene transcription in eukaryotic organisms (64, 65, 66). Intriguingly, the expression pattern of the FACT displays a robust correlation with that of the *Pf*NPM during the entire IDC (45, 67, 68). The observation led us to hypothesize the synergistic function of *Pf*NPM and FACT in chromatin organization, thereby exerting regulatory control over the transcriptional processes in trophozoite-stage parasites. Nevertheless, it is crucial to acknowledge that the assertion is speculative, and additional *in vitro* and *in vivo* experiments are needed to investigate this hypothesis.

*Pf*, a unicellular protozoan parasite, is a member of the apicomplexan phyla, including various other unicellular protozoan parasites. A protein named nuclear factor 3 (NF3), showing substantial sequence similarity to nucleoplasmins, has been reported in the apicomplexan parasite *Toxoplasma gondii*. *Tg*NF3 has been shown to interact with promoters of key genes pertaining to metabolism, transcription, translation, and cell division, thereby likely controlling the expression of various genes by modulating chromatin architecture in *T. gondii* (69). *Pf*NPM shares significant sequence identity (∼60%) with *Tg*NF3 (Fig. S12), thus raising the possibility that, in addition to its role as a histone chaperone, *Pf*NPM may interact with certain gene promoters to modulate the chromatin dynamics, thereby influencing transcriptional regulation. Further investigations are warranted to explore this potential avenue. To summarize, our structural and functional studies confirmed that the *Pf*NPM is a member of the nucleoplasmin family of histone chaperones. We propose that *Pf*NPM might function in combination with FACT histone chaperone for modulating the chromatin landscape.

## Experimental procedures

### Multiple sequence alignment

The multiple sequence alignments of *Pf*NPM NTD with other nucleoplasmins and *Pf*NPM with putative nucleoplasmins from other apicomplexan family members were performed using the T-Coffee server (70), and the representative images were prepared using the Esprit 4.0 server (71).

### Phylogenetic analysis

The phylogenetic analyses of nucleoplasmins were performed using MEGA11 software (https://www.megasoftware.net/) (72). In brief, the amino acid sequences of the nucleoplasmin domain of *Pf*NPM and other nucleoplasmins were aligned using the ClustalW software (http://www.ebi.ac.uk/clustalw/) (73), and a phylogenetic tree was constructed using the neighbour-joining method and a Dayhoff matrix-based model (74, 75).

### Cloning expression and purification

The total mRNA *of Pf* was used to synthesize full-length *Pf*NPM cDNA, which subsequently was used to prepare the *Pf*NPM constructs. The *Pf*NPM constructs, including *Pf*NPM full-length (residues 1–315), *Pf*NPM NTD (spanning residues 1–98), *Pf*NPM A1 (up to A1 acidic tract; residues 1–110), and *Pf*NPM A3 (up to A3 acidic tract; residues 1–170) were PCR amplified and cloned in-frame into a pET28a(+) vector between NcoI and XhoI restriction endonuclease sites for expression with a noncleavable C-terminal hexa-histidine tag. All clones were confirmed using colony PCR and DNA sequencing. The *Pf*NPM expression constructs—including *Pf*NPM NTD, *Pf*NPM A1, and *Pf*NPM A3—were transformed into *E*. *coli* BL21 DE3 codon plus RIL cells. The transformed bacterial cells were cultured in 2 × YT medium containing 25 μg/ml chloramphenicol and 100 μg/ml kanamycin at 37 °C. The induction of bacterial culture was done at 0.4 to 0.6 absorbance at 600 nm with 0.5 mM IPTG and incubated for 4 h at 37 °C. Subsequently, cells were harvested and resuspended in a lysis buffer [20 mM Tris (pH 7.5), 500 mM NaCl, 2 mM β-mercaptoethanol, 1 mM PMSF], and cell lysis was performed using a probe-type ultrasonic processor followed by centrifugation at 42,000*g* for 50 min at 4 °C. The supernatant was loaded on HisTrap FF 5 ml nickel affinity column (Cytiva), pre-equilibrated with the lysis buffer, and washed with the lysis buffer containing 10 mM imidazole. An elution buffer [20 mM Tris (pH 7.5), 300 mM NaCl, 2 mM β-mercaptoethanol, 300 mM imidazole] was used to elute the bound protein, which was further subjected to SEC using a HiLoad 16/600 Superdex 200 prep-grade column (Cytiva) at 4 °C in SEC buffer [20 mM Tris (pH 7.5), 150 mM NaCl, 2 mM β-mercaptoethanol]. The purified protein was concentrated using a 30 kDa cut-off centrifugal concentrator, flash-frozen in liquid nitrogen, and stored at −80 °C.

### Recombinant core histones

The codon-optimized constructs for the core histones H2A, H2B, H3, and H4 were a kind gift from Dr Curtis A. Davey (Nanyang Technological University). The expression of the core histones without a fusion tag from these constructs was performed as has been described before (76). In brief, the constructs in the pET21a(+) vector were individually transformed into *E. coli* BL21 (DE3) pLysS cells and grown in 2 × YT medium at 37 °C. The overexpression of the histones was induced with 0.4 mM IPTG when the absorbance at 600 nm of the culture reached 0.4, and the induction was allowed to proceed for 3 h at 37 °C. The individual histones were obtained as inclusion bodies and purified by SEC using a HiLoad 16/600 Superdex 200 prep-grade column under denaturing conditions. The purified histones were subjected to dialysis against double-distilled water, aliquoted into smaller volumes, lyophilized, and stored in a −80 °C freezer.

### Preparation of histone assemblies

The lyophilized core histones were dissolved in a denaturing buffer containing 7 M guanidinium hydrochloride, and the partner histones (H2A and H2B for H2A/H2B dimer or H3 and H4 for H3/H4 tetramer or all four core histones for histone octamer) were mixed in equimolar stoichiometry. The unfolded core histone mixtures were refolded to obtain H2A/H2B dimer, H3/H4 tetramer, and histone octamer by dialyzing against a refolding buffer containing 20 mM Tris (pH 7.5), 2 M NaCl, 1 mM EDTA, 10 mM β-mercaptoethanol, and 1 mM PMSF. The H2A/H2B dimer, H3/H4 tetramer, and histone octamer thus assembled were purified by SEC utilizing a HiLoad 16/600 Superdex 200 prep-grade column, pre-equilibrated with the refolding buffer. The peak fractions were pooled together, concentrated, and used for the various experiments.

### Nucleosome core particle reconstitution

After mixing the histone octamer and Widom’s 145 bp “601” DNA in TCS-2 buffer in an equimolar stoichiometry, the mixtures were dialyzed using a 7 kDa cut-off membrane against TCS-0.85 buffer containing 0.85 M KCl for 2.5 h. The concentration of KCl in the dialysis buffer was brought down to 0 KCl by decreasing it gradually over the course of five buffer changes (TCS-0.85, TCS-0.65, TCS-0.45, TCS-0.25, and TCS-0) at an interval of 2.5 h each. The gradual reduction in the salt concentration allows the stable assembly of 145 bp DNA over histone octamer, yielding the NCP. The quality of the reconstituted NCP was examined by subjecting it to 6% Native PAGE, followed by staining with SYBR Safe DNA gel stain and Coomassie Brilliant Blue R 250.

### Analytical SEC

All analytical SEC experiments were performed using a Superdex 200 Increase 10/300 Gl column (Cytiva) at 4 °C. The homogeneity of the purified *Pf*NPM NTD, *Pf*NPM A1, and *Pf*NPM A3 was examined by subjecting them to the analytical SEC at 4 °C in an SEC buffer [20 mM Tris (pH 7.5), 300 mM NaCl, 1 mM β-mercaptoethanol].

The thermal stability of *Pf*NPM NTD pentamer was examined by heating the samples at various temperatures from 20 °C to 100 °C and subjecting them to analytical SEC in the SEC buffer. Salt stability of the *Pf*NPM NTD was examined by analytical SEC in SEC buffer containing varying concentrations of NaCl (ranging from 150 mM to 2.0 M). Similarly, the urea stability of *Pf*NPM was examined using analytical SEC in SEC buffer containing increasing concentrations of urea (ranging from 1.0 M to 5.0 M). The protein samples were preincubated with respective urea concentrations containing SEC buffer for 16 h at room temperature before subjecting to SEC. The eluted fractions were analyzed on 18% SDS-PAGE after staining with Coomassie Brilliant Blue R 250.

### Analytical ultracentrifugation

The SV-AUC experiments to determine the molecular weight and other hydrodynamic parameters of *Pf*NPM proteins and their histone complexes were conducted using Optima AUC analytical ultracentrifuge (Beckman Coulter). The absorbance values at 280 nm of protein samples were kept between 0.2 and 0.5. SEDNTERP software (http://www.jphilo.mailway.com/sednterp.htm) (77) was used to estimate the density and viscosity of the buffer and the partial specific volume of the protein. SEDFIT software (https://sedfitsedphat.github.io/) (78) was used to process the raw data using a continuous size distribution model. GUSSI software (https://www.utsouthwestern.edu/research/core-facilities/mbr/software/) (77) was utilized for figure preparation.

### Isothermal titration calorimetry

The *Pf*NPM and assembled H2A/H2B and H3/H4 were dialyzed overnight at 4 °C into a buffer containing 20 mM Tris (pH 7.4), 300 mM NaCl, and 1 mM β-mercaptoethanol to perform the ITC experiment. All ITC experiments were conducted at 20 °C with the help of a MicroCal PEAQ-ITC machine (Malvern Panalytical). The titration of 100 to 250 μM of *Pf*NPM A1 and *Pf*NPM A3 from the syringe into the cell containing 10 to 30 μM of either assembled H2A/H2B dimer or H3/H4 tetramer was conducted, and the heat change was recorded. The heat change from the *Pf*NPM histone interactions was then substrated by the heat of dilution obtained by titrating *Pf*NPM into the buffer, the buffer into the histone assemblies, and the buffer into the buffer. The PEAQ-ITC analysis software (https://www.malvernpanalytical.com/en/support/product-support/software/microcal-peaq-itc-analysis-software-v141) was utilized to fit the subtracted data points into the one-site binding model, and the dissociation constant (KD), the enthalpy (ΔH), the entropy (ΔS), and the stoichiometry of interaction were examined.

### Small-angle X-ray scattering

All the SAXS experiments for *Pf*NPM NTD, *Pf*NPM A1, *Pf*NPM A3, and *Pf*NPM A3 histone complexes were conducted at the BM29 BioSAXS beamline of the European Synchrotron Radiation Facility (Grenoble, France) equipped with a Pilatus 1 M detector. The averaging and buffer subtraction were performed at the pipeline developed at the beamline. The ATSAS program (https://www.embl-hamburg.de/biosaxs/software.html) suite (79) was employed to plot and fit SAXS curves and to obtain all structural parameters and bead models. Briefly, the *Rg*, maximum particle dimension (Dmax), pair distribution function (P(r)), and the excluded particle volume (Vp) were estimated using GNOM (https://www.embl-hamburg.de/biosaxs/gnom.html). DAMMIF (https://www.embl-hamburg.de/biosaxs/dammif.html) was employed to generate 10 low-resolution bead models, then DAMAVER (https://www.embl-hamburg.de/biosaxs/damaver.html) aligned them and generated an averaged bead model (80). The *Pf*NPM A1 and *Pf*NPM A3 structure models were generated using the Galaxy Homomer server (https://galaxy.seoklab.org/cgi-bin/submit.cgi?type=HOMOMER) (81). FoXS online server (https://modbase.compbio.ucsf.edu/foxs/) (82) was employed to compare the experimental scattering with the theoretical scattering from structure models. FoXSDock online server (https://modbase.compbio.ucsf.edu/foxsdock/) (82) was employed to obtain the best-fit structure model of the complexes to the experimental scattering. PyMOL (Schrödinger, LLC) software (https://www.pymol.org/) was utilized for image preparation of the SAXS structures.

### Cryo-EM grid preparation and image processing

3.5 μl of the purified *Pf*NPM A3 (1.5 mg/ml) was dispensed on a freshly plasma-cleaned Quantifoil copper (1.2/1.3, 300 mesh) grid afterglow discharge for 1 min. The Vitrobot Mark IV instrument (FEI/Thermo Fisher Scientific) was utilized for grid preparation (blot time 5 s, blot force 5, wait time 10 s, single blot) at 100% humidity and 4 °C temperature immediately before plunge freezing in liquid ethane. A total of 4679 movies were collected using EPU software (https://www.thermofisher.com/in/en/home/electron-microscopy/products/software-em-3d-vis/epu-software.html) on a 300 kV Titan Krios microscope equipped with a K2 summit direct electron detector in super-resolution mode (0.4226 Å/pixel) at a magnification of 165,000. The exposure time for each movie was 7 s with 50 frames and a total dose of 58 e−/Å2. Data collection was performed within a defocus range of −0.8 to −1.6. Motion correction and CTF estimation were performed in Relion 3.1 (https://relion.readthedocs.io/en/release-3.1/). Subsequently, the micrographs were transferred to CryoSPARC for further processing. A small subset of micrographs was used for manual particle picking. The manually picked particles were further used for template-based particle picking, which resulted in 1,069,357 particles. Laplacian-of-Gaussian–based picking resulted in 1,276,908 particles. Usage of the conventional neural network-trained Topaz particle-picking pipeline yielded 921,112 particles. Further, iterative rounds of 2D classification were performed.

### Crystallization, data collection, and data processing

*Pf*NPM NTD was used for crystallization screening at a 57 mg/ml concentration in a buffer containing 20 mM Tris (pH 7.5), 150 mM NaCl, and 1 mM β-mercaptoethanol. Multiple crystals of *Pf*NPM NTD were obtained in a PEG ion crystallization screening condition having 0.2 M sodium malonate (pH 5.0) and 20% PEG3350. A single crystal soaked in the screening condition supplemented with 20% ethylene glycol as a cryoprotectant diffracted to 3.25 Å. *Pf*NPM NTD crystal diffraction data were collected at the European Synchrotron Radiation Facility ID30-A3 (MASSIF-3) beamline and recorded on an Eiger X 4M detector from Dectris. The diffraction data were processed using iMosflm followed by AIMLESS from the CCP4 (https://www.ccp4.ac.uk/) suite (83). The crystal belonged to the trigonal space group P 3_1_ 2 1 with unit cell dimensions a = b = 89.35 Å, c = 113.28 Å, α = β = 90°, and γ = 120°. The crystal structure of *Pf*NPM NTD was obtained using the Phenix Phaser molecular replacement program using a monomer of *At*FKBP53 NTD (PDB ID 6J2Z; chain A) as a model and searching for five copies. Five molecules were present in one asymmetric unit. Crystallographic refinement and model building were conducted iteratively using COOT (https://www2.mrc-lmb.cam.ac.uk/personal/pemsley/coot/) and Phenix.Refine (https://phenix-online.org/documentation/reference/refine_gui.html) (84, 85). Non-crystallographic symmetry and translation-libration-screw parameters were used during the refinement steps. The stereochemical quality of the structure was checked using the MolProbity (https://molprobity.biochem.duke.edu/) program (86). PyMOL was used to prepare the structure figures and for structural superpositions.

### Pull-down assays

To check for the interaction of *Pf*NPM NTD, *Pf*NPM A1, and *Pf*NPM A3 with H2A/H2B and H3/H4, 5 μM of His-tagged *Pf*NPM (*Pf*NPM NTD, *Pf*NPM A1, and *Pf*NPM A3) was mixed separately with 20 μM of assembled H2A/H2B or H3/H4. The samples were then incubated with nickel-nitrilotriacetic acid affinity resin beads (Clontech) pre-equilibrated with a buffer containing 20 mM Tris (pH 7.5), 300 mM NaCl, 40 mM imidazole (pH 7.5), 10 μg/ml bovine serum albumin, and 1 mM β-mercaptoethanol for 30 min at 4 °C. The incubated beads were then washed with 10 column volumes of wash buffer [20 mM Tris (pH 7.5), 300 mM NaCl, 50 mM imidazole (pH7.5), 0.2% Tween-20, 1 mM β-mercaptoethanol]. The complexes were eluted from the beads with elution buffer [20 mM Tris (pH 7.5), 300 mM NaCl, 500 mM imidazole (pH 7.5), 1 mM β-mercaptoethanol]. The eluted samples were analyzed on 18% SDS-PAGE after staining with Coomassie Brilliant Blue R 250.

The stability of *Pf*NPM A1 and *Pf*NPM A3 complexes with assembled H2A/H2B and H3/H4 in the presence of salt was also studied using a pull-down assay using nickel-nitrilotriacetic acid affinity resin beads. Toward this end, the complexes were incubated with the beads in individual reactions with buffers containing increasing NaCl concentrations of 0.3 M to 1 M. Further, the beads were washed, and the proteins were eluted and analyzed on an 18% SDS-PAGE.

To examine the binding preference of *Pf*NPM for H2A/H2B and H3/H4, 5 μM of *Pf*NPM A1 and *Pf*NPM A3 were mixed with pre-equilibrated nickel-nitrilotriacetic acid affinity resin beads, and subsequently, 20 μM of assembled H3/H4 was mixed and incubated for 30 min at 4 °C. Next, the bound beads were washed with 5 ml of wash buffer followed by equilibration with 5 ml of equilibration buffer. Subsequently, the equilibrated beads were mixed with 20 μM of assembled H2A/H2B and incubated for 30 min at 4 °C. Similarly, the pull-down assay was carried out by reversing H2A/H2B and H3/H4 in the reaction. Finally, the bound proteins were eluted from the beads using the elution buffer and loaded on 18% SDS-PAGE gel for analysis.

### Electrophoretic mobility shift assay

To study the binding of *Pf*NPM with NCP, purified *Pf*NPM NTD, *Pf*NPM A1, and *Pf*NPM A3 were mixed with reconstituted NCP in a buffer containing 20 mM Tris (pH 7.5), 50 mM NaCl, 1 mM EDTA and 1 mM DTT. *At*FKBP53 NTD was used as a positive control in this assay. The mixtures were incubated at 4 °C for 1 h and subjected to electrophoresis on a 6% Native PAGE in 0.5 × Tris borate EDTA buffer for 180 min at 60 V, and the gel was stained with SYBR Safe DNA gel stain, followed by Coomassie Brilliant Blue R 250 for visualization of DNA and protein components of the samples, respectively.

### Nucleosome assembly assay

To investigate the nucleosome assembly function of *Pf*NPM, a plasmid supercoiling assay was performed. Five hundred micrograms of supercoiled pUC19 plasmid was relaxed by treatment with 0.5 U of wheat germ topoisomerase I (Inspiralis) at 37 °C for 1 h. Simultaneously, 5 μM each of assembled H2A/H2B dimer and H3/H4 tetramer were mixed with 2 μM of *Pf*NPM NTD, *Pf*NPM A1, or *Pf*NPM A3 in a buffer comprised of 20 mM Tris (pH 7.5), 100 mM NaCl, 1 mM MgCl_2_, 0.5 mM DTT, and 0.1 mg/ml bovine serum albumin and incubated at 30 °C for 30 min. Subsequently, the relaxed pUC19 plasmid was mixed with the preincubated *Pf*NPM, H2A/H2B, and H3/H4 mixture and incubated at 37 °C for 1 h. Finally, the pUC19 plasmid was extracted using a phenol-chloroform extraction method and subsequently subjected to 1% agarose gel electrophoresis, followed by visualization by SYBR Safe DNA gel stain.

### *Pf* culture

*Pf* strain 3D7 strain culture was maintained at 37 °C in RPMI-1640 medium supplemented with 0.5% AlbuMAX-II, using RBC of 5% hematocrit under 90% N_2_ and 5% CO_2_. The parasite was harvested and resuspended in a buffer containing 50 mM Tris (pH 8.0), 150 mM NaCl, and protease inhibitor cocktail (Roche).

### Western blotting

The *Pf* cells were dissolved in RIPA buffer and kept at 4 °C for 1 h. Subsequently, the cells were lysed using a water bath sonicator at 4 °C. The lysate samples were then subjected to 18% SDS-PAGE and transferred onto a polyvinylidene fluoride membrane. The polyvinylidene fluoride membrane was blocked for 1 h at room temperature with 5% skimmed milk in Tris-buffered saline with Tween-20 buffer. The membranes were probed with anti-*Pf*NPM antibodies (1:1000) and subsequently probed with horseradish peroxidase–conjugated secondary antibodies (1:2500) and visualized by enhanced chemiluminescence. Custom produced anti-*Pf*NPM antibodies were validated utilizing ELISA.

### IF microscopy

The blood-stage parasites in ring, trophozoite, and schizont stages were washed with PBS and fixed using 4% paraformaldehyde and 0.0075% glutaraldehyde, followed by permeabilization with 0.1% Triton X-100 and treatment with 0.1 M glycine. The permeabilized cells were blocked with 2% bovine serum albumin in PBS and incubated with anti-*Pf*NPM antibodies (1:1000) in blocking buffer for 4 h, followed by Alexa Flour 488–conjugated secondary antibodies (1:400) for 2 h. After washing with PBS, the cells were incubated with 4′,6-diamidino-2-phenylindole (1 μg/ml) for nuclear staining. Finally, the images were captured using an Olympus IX83 inverted microscope equipped with a DP73 high-performance camera.

## Data availability

Atomic coordinates and structure factor of *Pf*NPM crystal structure have been deposited with the PDB accession ID 8ZS0. SAXS data of *Pf*NPM NTD (1–98), *Pf*NPM (1–110), *Pf*NPM (1–170), *Pf*NPM (1–170)–H2A/H2B complex, and *Pf*NPM (1–170)–H3/H4 complex have been deposited with SASBDB accession IDs SASDVQ2, SASDVR2, SADVS2, SASDVT2, and SASDVU3, respectively.

## Supporting information

This article contains supporting information (87).

## Conflict of interest

The authors declare that they have no conflicts of interest with the contents of this article.
