# Supplementary material for: Structure-function studies of a nucleoplasmin isoform from Plasmodium falciparum
Source: J Biol Chem. 2025 Mar 4;301(4):108379. doi: 10.1016/j.jbc.2025.108379 (PMC11993163; doi:10.1016/j.jbc.2025.108379)
Supplement: Supporting information [file mmc1.docx]

**SuppORTING INFORMATION**

**Structure-function studies of a nucleoplasmin isoform from *Plasmodium falciparum***

Ketul Saharan^1,2^, Somanath Baral^1^, Surajit Gandhi^1,2^, Ajit Kumar Singh^1^, Sourav Ghosh^3^, Rahul Das^2,3^, Viswanathan Arun Nagaraj^3^, Dileep Vasudevan^1,4,#^

^1^Structural Biology Laboratory, BRIC-Institute of Life Sciences (BRIC-ILS), Bhubaneswar - 751023, India.

^2^Regional Centre for Biotechnology, Faridabad - 121001, India.

^3^Malaria Parasite Biology Laboratory, BRIC-Institute of Life Sciences (BRIC-ILS), Bhubaneswar - 751023, India.

^4^Structural Biology Laboratory, BRIC-Rajiv Gandhi Centre for Biotechnology (BRIC-RGCB), Thiruvananthapuram - 695014, India.

^#^Correspondence to: Dileep Vasudevan, E-mail: [dvasu@rgcb.res.in](mailto:dvasu@rgcb.res.in)

**Running title:** Characterization of *Plasmodium falciparum* nucleoplasmin

Supplementary Figures 1-12

Supplementary Tables 1-5

Supplementary Reference 1

**Supplementary Figures:**


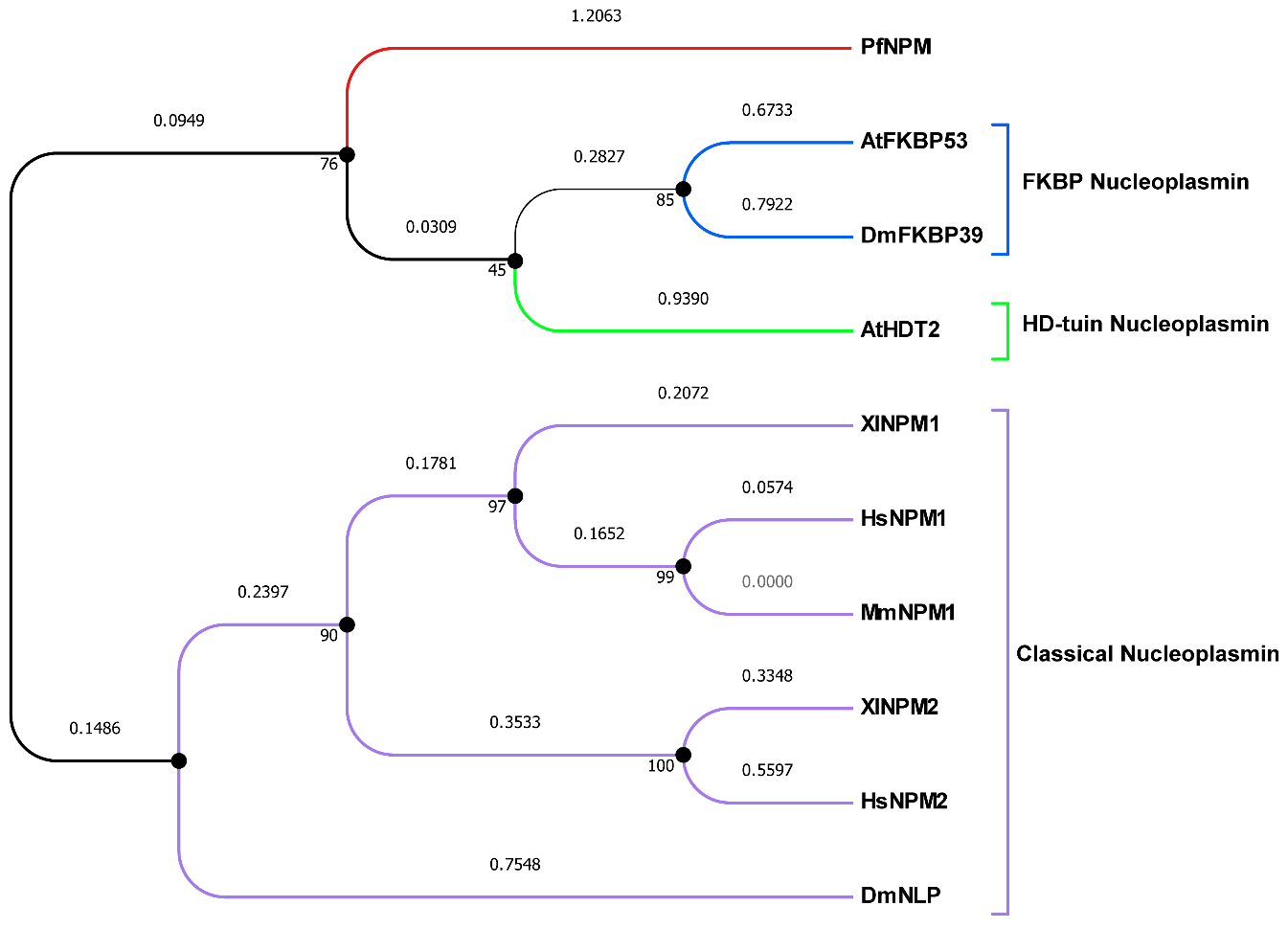


**Supplementary Figure 1. Phylogenetic analysis of *Pf*NPM and other nucleoplasmins.** Full-length nucleoplasmin sequences were used to generate the evolutionary tree. The Neighbour Joining method using Dayhoff matrices was used to calculate the evolutionary distance for the tree. The numbers at the nodes reflect the bootstrapping value from 1000 repetitions [1], while the numbers above each horizontal branch show evolutionary changes measured in genetic variation.


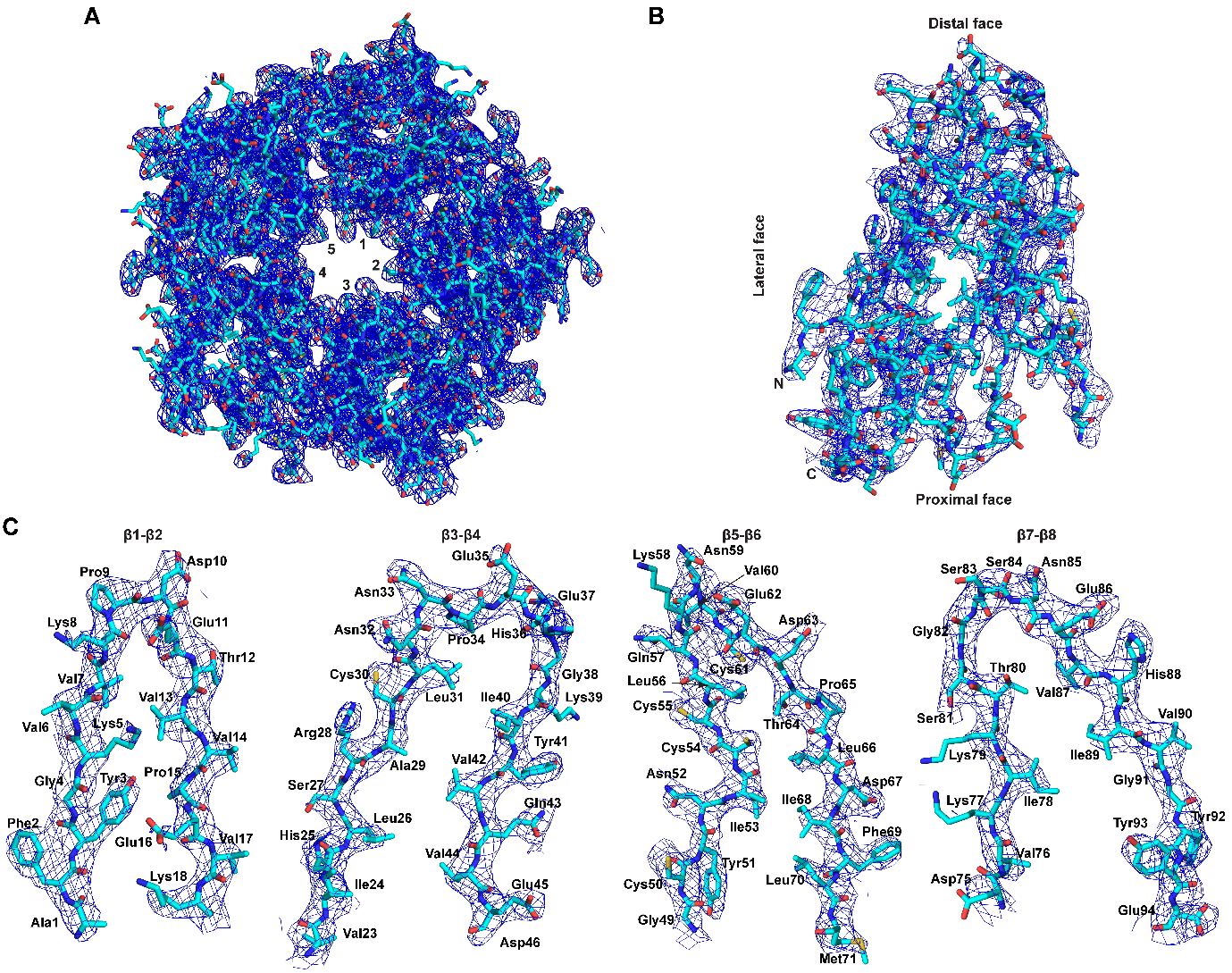


**Supplementary Figure 2. Electron density map of *Pf*NPM crystal structure. The final (2Fo-Fc) electron density map (contoured at 1 σ, blue) fitted with (A) *Pf*NPM pentamer, (B) PfNPM monomer, and (C) the β strands β1 to β2, β3 to β4, β5 to β6 and β7 to β8 shown in stick model. The map was generated using COOT.**


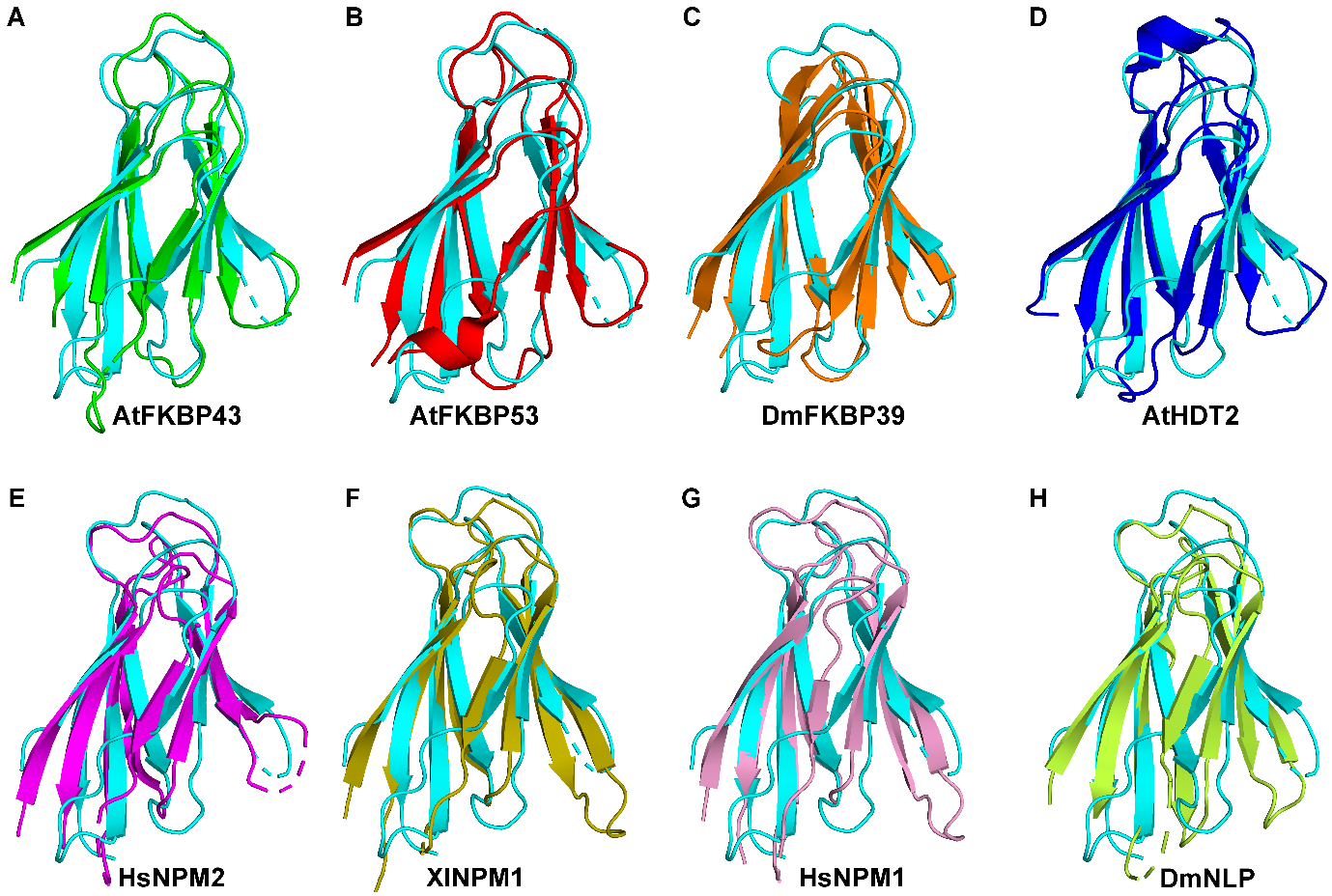


**Supplementary Figure 3. Structural comparison of *Pf*NPM NTD with other nucleoplasmins.** Cartoon representation for the structural alignment of *Pf*NPM NTD monomer (in cyan) versus **(A)** *At*FKBP43 NTD (green; PDB ID: 7WIM) (RMSD, 0.813 Å), (**B)** *At*FKBP53 NTD (red; PDB ID: 6J2Z) (RMSD, 1.336 Å), **(C)** *Dm*FKBP39 NTD (orange; PDB ID: 4CA9) (RMSD, 1.234 Å), **(D)** *At*HDT2 NTD (blue; PDB ID: 7VMF) (RMSD, 1.834 Å), (**E)** HsNPM2 (violet; PDB ID: 3T30) (RMSD, 1.081 Å), **(F)** *Xl*NPM1 NTD (olive; PDB ID: 1XE0) (RMSD, 1.564 Å), (**G)** *Hs*NPM1 (light pink; PDB ID: 2B1P) (RMSD, 1.216 Å) and **(H)** *Dm*NLP (light green; PBD ID: 1NLQ) (RMSD, 1.518 Å). The alignments were performed using PyMOL. To refine the structural alignment and minimize deviations, backbone-driven optimization was implemented through the 'super' command in PyMOL.


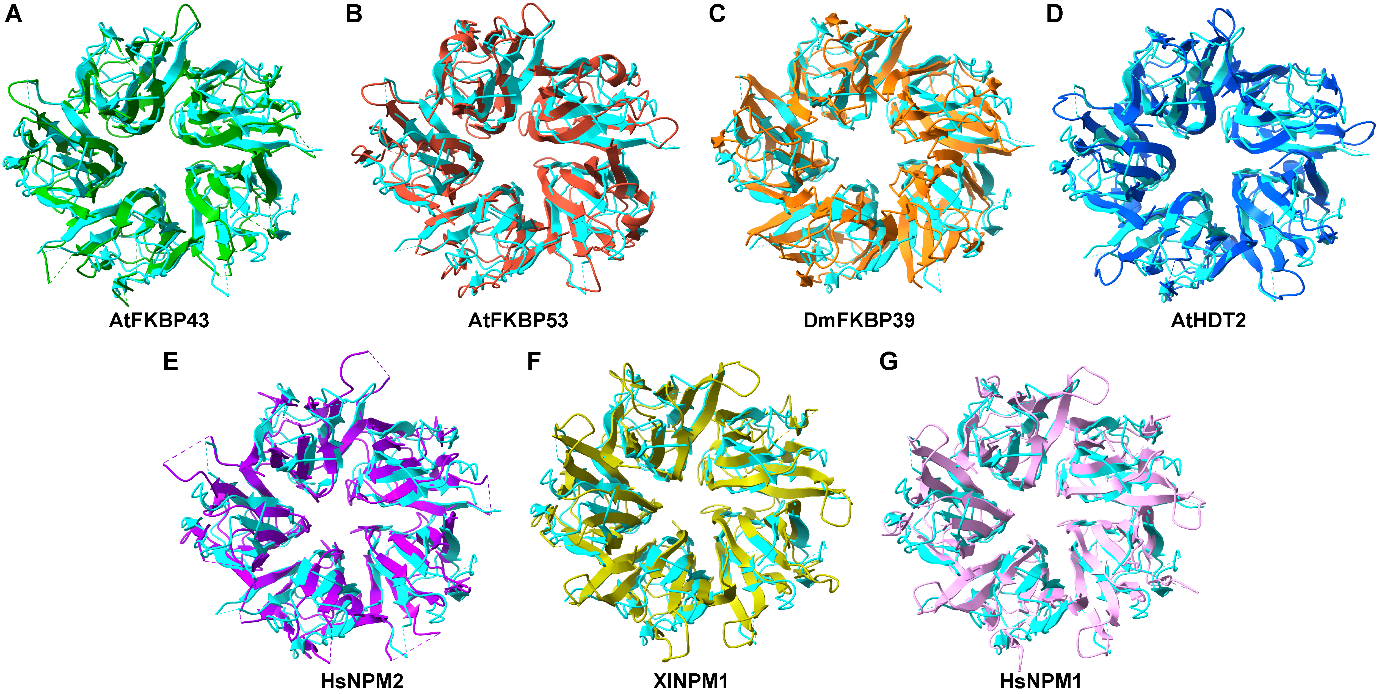


**Supplementary Figure 4. Structural comparison of *Pf*NPM NTD pentamer with other nucleoplasmins pentamer.** Cartoon representation for the structural alignment of *Pf*NPM NTD pentamer (in cyan) versus the pentamer of **(A)** *At*FKBP43 NTD (green; PDB ID: 7WIM) (RMSD, 0.941 Å), (**B)** *At*FKBP53 NTD (red; PDB ID: 6J2Z) (RMSD, 1.084 Å), **(C)** *Dm*FKBP39 NTD (orange; PDB ID: 4CA9) (RMSD, 1.091 Å), **(D)** *At*HDT2 NTD (blue; PDB ID: 7VMF) (RMSD, 0.974 Å), (**E)** *Hs*NPM2 (violet; PDB ID: 3T30) (RMSD, 1.317 Å), **(F)** *Xl*NPM1 NTD (olive; PDB ID: 1XE0) (RMSD, 1.246 Å), (**G)** *Hs*NPM1 (light pink; PDB ID: 2P1B) (RMSD, 1.325 Å). The alignments were performed using ChimeraX. The 'SS' option in MatchMaker (ChimeraX) was used to define the pairing of corresponding chains within each structure explicitly during the alignment.


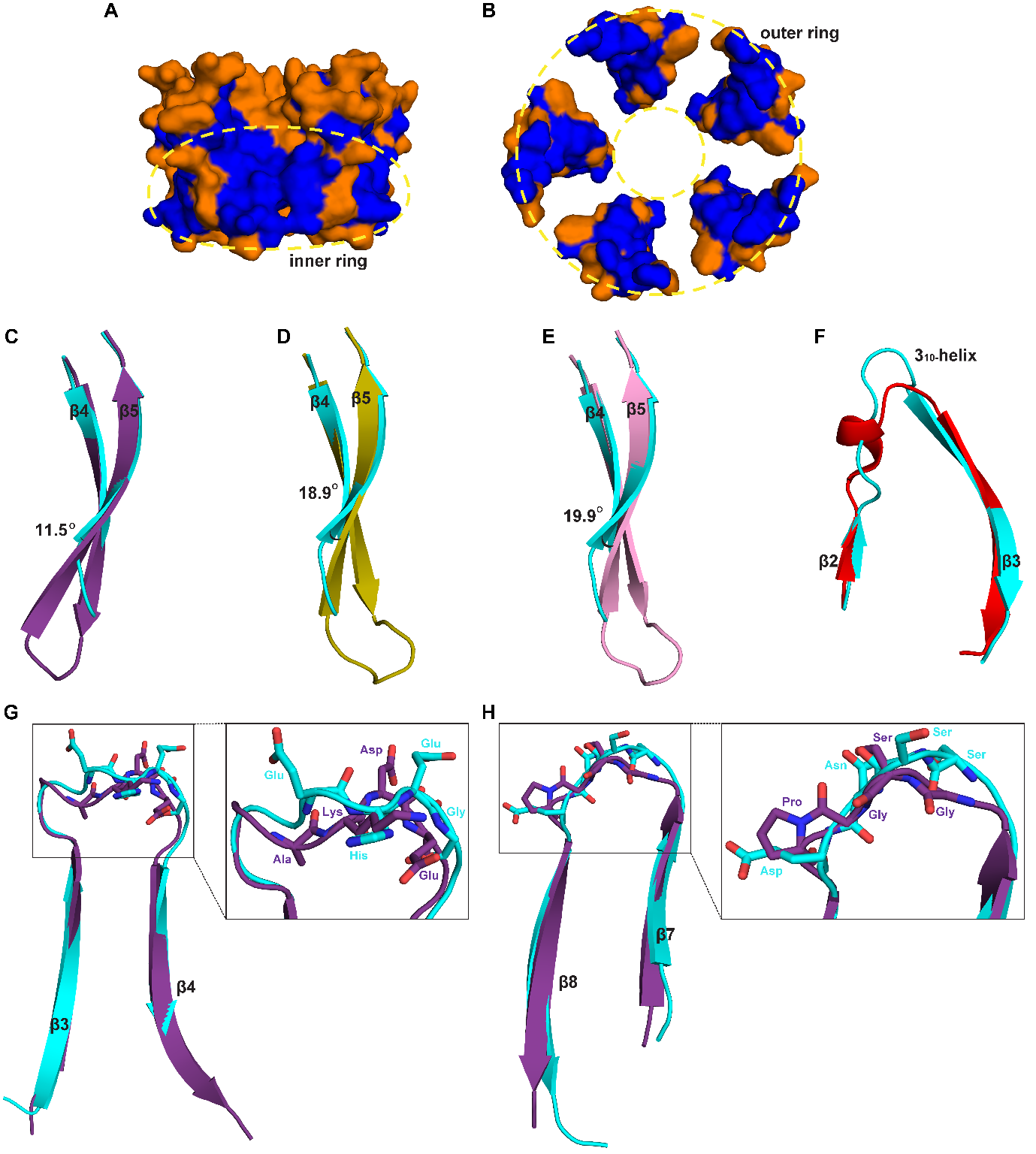


**Supplementary Figure 5. Structural details of *Pf*NPM NTD and comparison with other nucleoplasmins.**The distribution of apolar residues across **(A)** the inner core, constituted by β-sheet 1, and **(B)** the outer core, constituted by β-sheet 2, as depicted in the surface representation with blue color. The polar residues are illustrated in orange color. **(C)**Structural alignment of β-hairpin of *Pf*NPM NTD (cyan) with *Xl*NPM2 (PDB ID: 1K5J; violet). The β-hairpin of *Xl*NPM2 protrudes 11.5° from the five-fold symmetry axis compared to *Pf*NPM. **(D)**Structural alignment of β-hairpin of *Pf*NPM NTD (cyan) with *Xl*NPM1 (PDB ID: 1XE0; light green). The β-hairpin of *Xl*NPM2 protrudes 18.9° from the five-fold symmetry axis compared to *Pf*NPM NTD. **(E)**Structural alignment of β-hairpin of *Pf*NPM NTD with *Hs*NPM1 (PDB ID: 2P1B; pink). The β-hairpin of *Hs*NPM1 protrudes 19.9° from the five-fold symmetry axis compared to *Pf*NPM NTD. **(F)** Structural alignment of Loop 2 between *Pf*NPM NTD and *At*FKBP53 NTD. AtFKBP53 NTD has a 3_10_ helix in the Loop 2, while PfNPM lacks it. **(G)**The structural comparison of Loop 3 between *Pf*NPM NTD (cyan) and *Xl*NPM2 (violet). *Xl*NPM2 possesses an AKDE motif on Loop 3, while *Pf*NPM has it as residues EHEG. **(H)** Structural comparison of Loop 8 between *Pf*NPM NTD (cyan) and *Xl*NPM2 (violet). *Xl*NPM2 possesses a GSGP motif on Loop 3, while *Pf*NPM has residues SSNE.


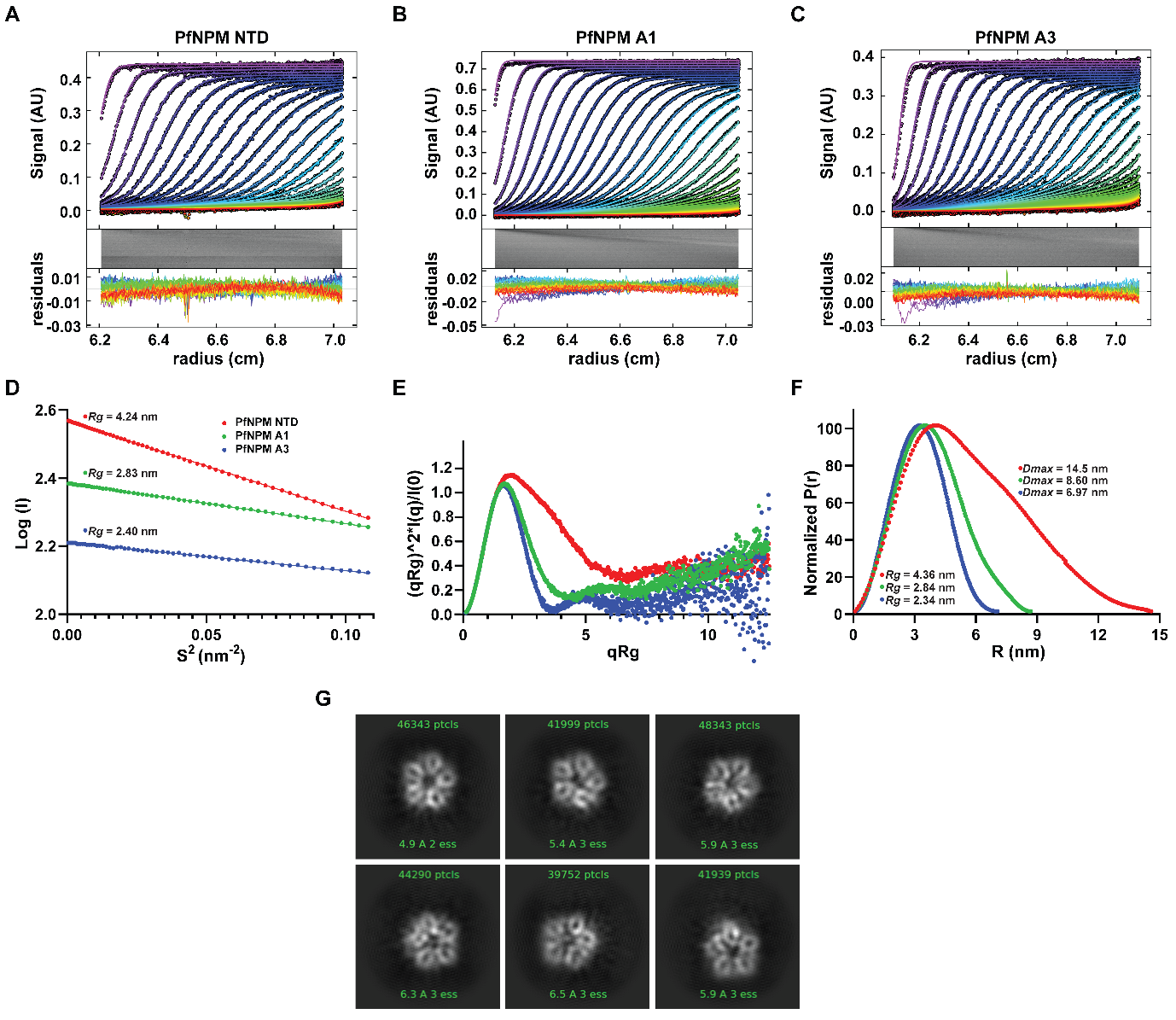


**Supplementary Figure 6. In-solution oligomeric state analysis of *Pf*NPM.**The SV-AUC experiment shows an overlay of the fitted and experimental curve (upper panel) and the residual plot (lower panel) for **(A)***Pf*NPM NTD,**(B)** *Pf*NPM A2, and **(C)** *Pf*NPM A3.**(D)**SAXS generated Guinier plot of *Pf*NPM NTD, *Pf*NPM A1, and *Pf*NPM A3, showing the linear fit of the experimental intensity data to the scattering at the low Q region. The profile shows a homogeneous distribution of protein samples with no indication of aggregation. **(E)**SAXS-generated Kratky plot of *Pf*NPM NTD, *Pf*NPM A1, and *Pf*NPM A3. *Pf*NPM NTD and *Pf*NPM A1 profiles suggest a completely globular nature, whereas *Pf*NPMA3 appears to have some unstructured regions as well. **(F)** The paired distance distribution plot for *Pf*NPM NTD, *Pf*NPM A1, and *Pf*NPM A3 obtained from SAXS data analysis shows a difference in the dimension of these proteins. **(G)**Cryo-EM-generated 2D class averages showing the proximal/distal faces of the pentameric *Pf*NPM A3 molecule.


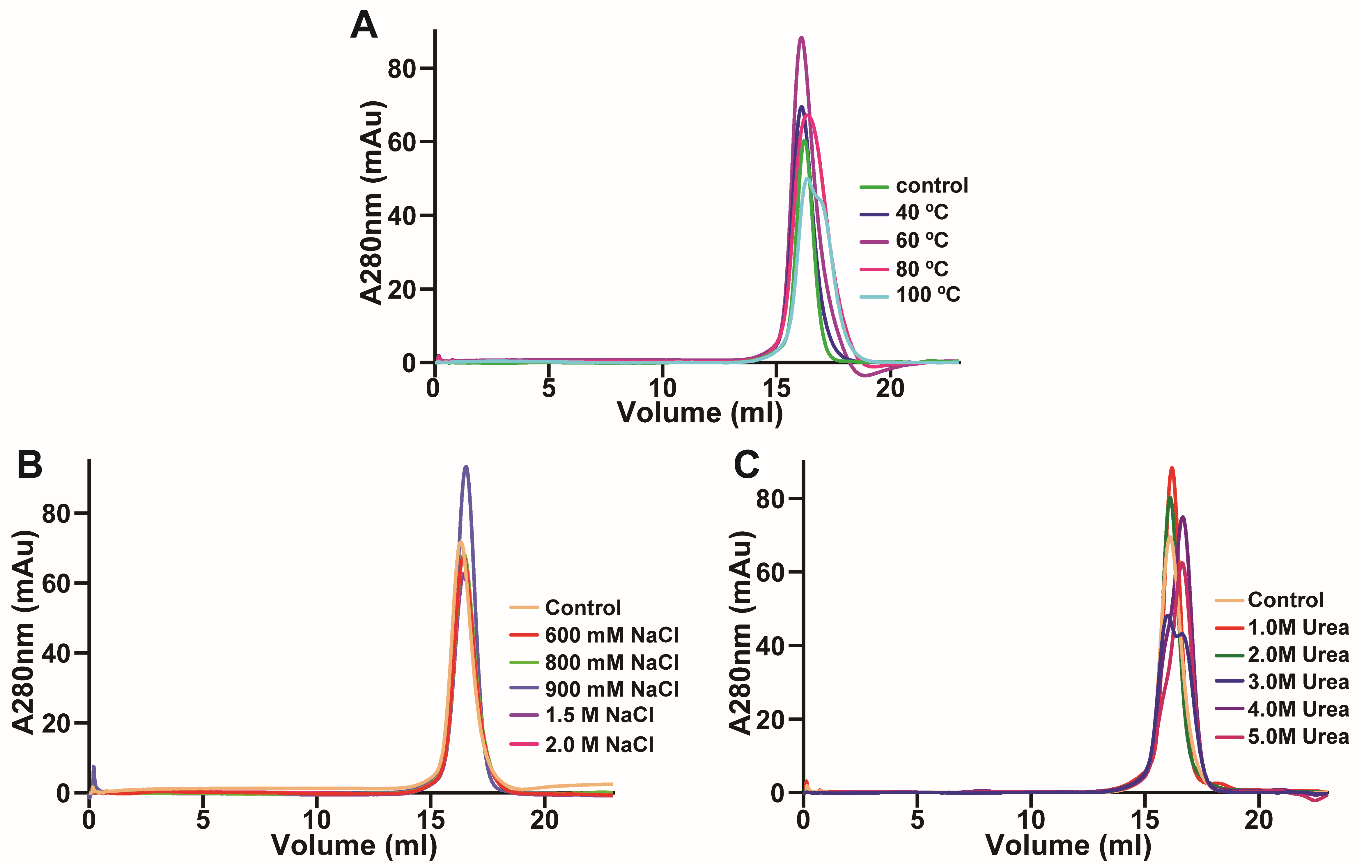
 **Supplementary Figure 7. Thermal and chemical stability analysis of *Pf*NPM NTD pentamer using Analytical SEC. (A)** Thermal stability analysis of *Pf*NPM NTD upon incubation at 40 °C to 100 °C temperature. *Pf*NPM NTD pentamer displayed stability up to 60 °C. (**B)** Salt stability analysis of PfNPM NTD pentamer with buffer containing increasing NaCl concentration from 600 mM to 2.0 M. *Pf*NPM NTD pentamer displayed stability up to 2.0 M NaCl (**C)** Urea stability analysis of *Pf*NPM NTD pentamer with buffer containing increasing urea concentration from 1.0 M to 5.0 M. *Pf*NPM NTD pentamer showed stability up 2.0 M urea.


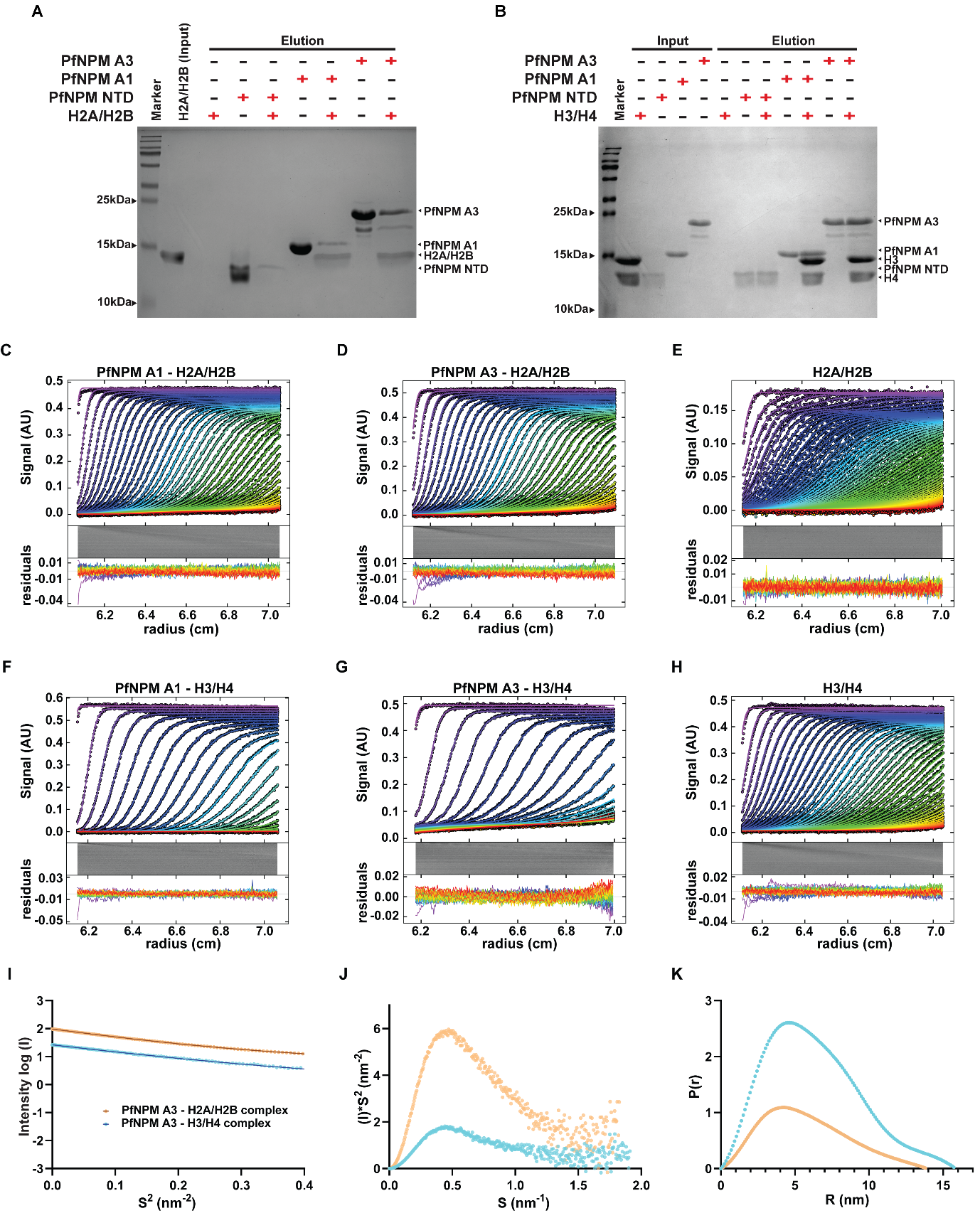


**Supplementary Figure 8. Interaction analysis of *Pf*NPM with assembled H2A/H2B and H3/H4. (A) P**ull-down assay of *Pf*NPM for its interaction with H2A/H2B dimer. The elution fractions recovered from the pull-down assay were subjected to an 18% SDS-PAGE and stained with Coomassie Brilliant Blue R 250. *Pf*NPM A1 and *Pf*NPM A3 showed H2A/H2B binding; however, *Pf*NPM NTD did not. **(B) P**ull-down assay of *Pf*NPM for its interaction with H3/H4 tetramer. The elution fractions recovered from the pull-down assay were subjected to an 18% SDS-PAGE and stained with Coomassie Brilliant Blue R 250. *Pf*NPM A1 and *Pf*NPM A3 showed H3/H4 binding; however, *Pf*NPM NTD did not. The overlay of the fitted vs. experimental curve (upper panel) and the residual plot (lower panel) obtained from the SV-AUC experiments conducted for **(C)** *Pf*NPM A1 – H2A/H2B complex, **(D)** *Pf*NPM A3 – H2A/H2B complex, **(E)** H2A/H2B dimer, **(F)** *Pf*NPM A1 – H3/H4 complex, **(G)** *Pf*NPM A3 – H3/H4 complex, and **(H)** H3/H4 tetramer. **(I)**Guinier plot for *Pf*NPM A3 – H2A/H2B complex and *Pf*NPM A3 – H3/H4 complex was obtained from SAXS analysis. Data points coinciding well with straight lines suggest that the samples are homogeneous.**(J)**The Kratky plot for *Pf*NPM A3 – H2A/H2B complex and *Pf*NPM A3 – H3/H4 complex obtained from SAXS analysis demonstrates a Gaussian curve indicative of the folded nature of the complexes. **(K)**The SAXS obtained distance distribution plots for the *Pf*NPM A3 – H2A/H2B and the *Pf*NPM A3 – H3/H4 complexes demonstrate the dimension of the complexes in nanometers.


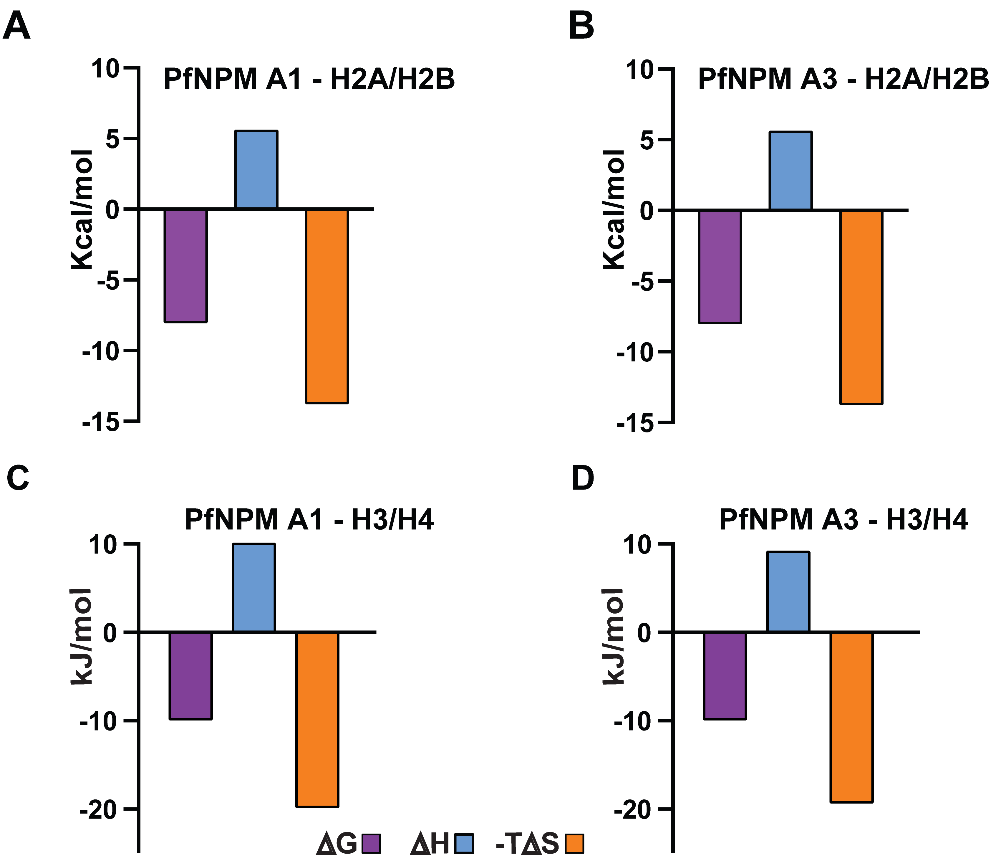


**Supplementary Figure 9. Isothermal titration calorimetry for the interaction of *Pf*NPM with H2A/H2B and H3/H4.** The signature plots for the titration of **(A)** assembled H2A/H2B into *Pf*NPM A1, **(B)** assembled H2A/H2B into *Pf*NPM A3, **(C)** assembled H3/H4 into *Pf*NPM A1, and **(D)** assembled H3/H4 into *Pf*NPM A3. The thermodynamic parameters such as ΔG (violet), ΔH (blue), and -TΔS (orange) are illustrated as bars.

**
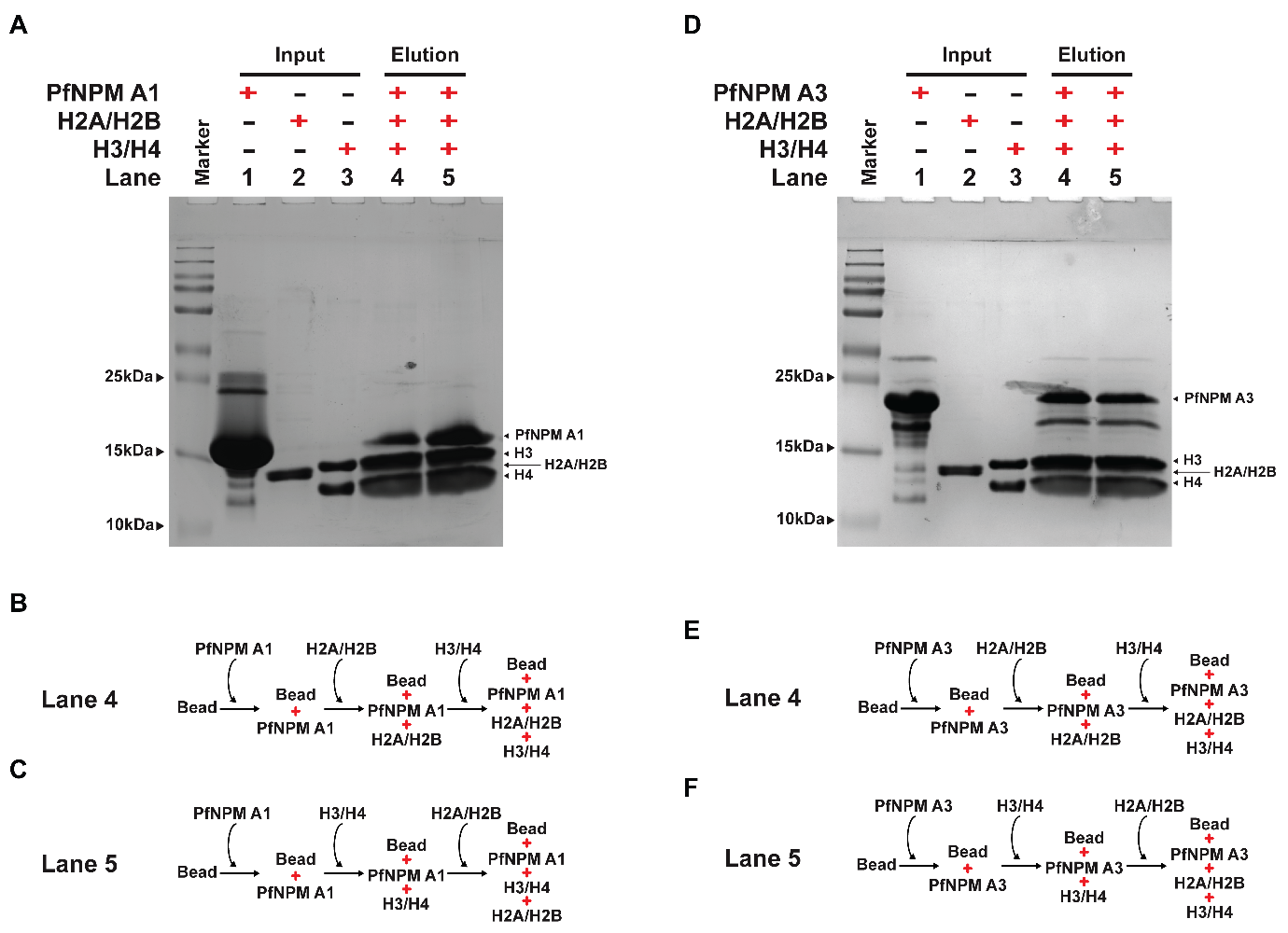
**

**Supplementary Figure 10. Competitive pull-down assay for *Pf*NPM interaction with H2A/H2B and H3/H4. (A**) The competitive pull-down assay for *Pf*NPM A1 histone complexes. The samples were subjected to 18% SDS-PAGE after Ni-NTA elution. The depicted lanes 1, 2, and 3 represent the input samples of *Pf*NPM A1, H2A/H2B, and H3/H4. The methodology employed for lanes 4 and 5 for the Ni-NTA experiment is illustrated in **(B)**and (**C)**, respectively.**(D)** The competitive pull-down assay for *Pf*NPM A3 histone complexes. The samples were subjected to 18% SDS-PAGE after Ni-NTA elution. The depicted lanes 1, 2, and 3 represent the input samples of *Pf*NPM A3, H2A/H2B, and H3/H4. The methodology employed for lanes 4 and 5 for the Ni-NTA experiment is illustrated in **(E)**and (**F)**, respectively. Overall, these results suggest that both H2A/H2B dimer and H3/H4 tetramer share the interaction site on *Pf*NPM, and, owing to its stronger binding affinity, H3/H4 tetramer substitutes the H2A/H2B dimer competitively.


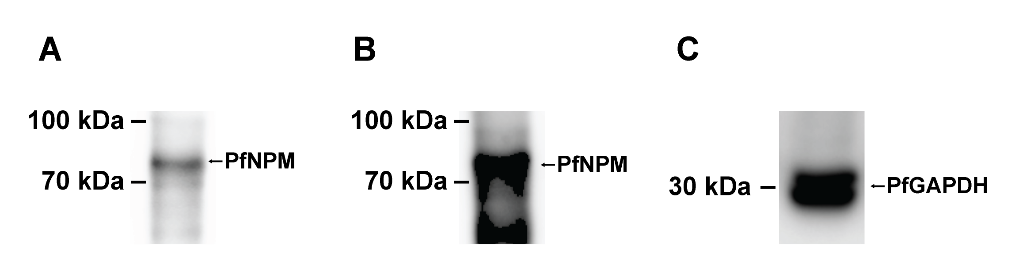


**Supplementary Figure 11. Western blot analysis of PfNPM in the blood stages of the parasite. (A)** Pf lysate probed using anti-*Pf*NPM antibodies. Anti-*Pf*NPM resulted in a band between 70 to 100 kDa size, higher than the theoretical molecular (35 kDa) of the full-length *Pf*NPM. **(B)** The same PVDF membrane stripped and probed with anti-Pan phospho (serine and threonine) antibodies. Anti-Pan phospho antibodies yielded overlapping bands to Anti-*Pf*NPM antibodies, demonstrating that the native *Pf*NPM within the parasite is phosphorylated. **(C)** Subsequently, the PVDF membrane was stripped again and probed with anti-GAPDH antibodies.


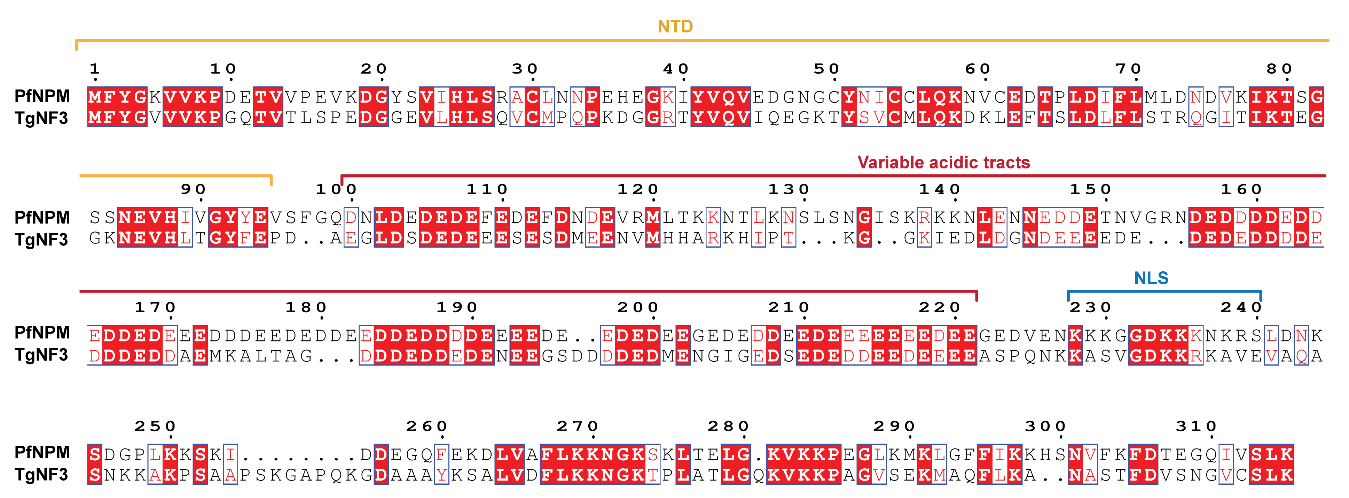


**Supplementary Figure 12. Sequence alignment of *Pf*NPM with *Toxoplasma gondii* NF3. Sequence comparison of** *Pf*NPM (UniProtKB ID: COH4U4) with *Tg*NF3 (UniProtKB ID: A0A7J6K2Z4) performed using ClustalW. The red boxes indicate the conserved residues among both proteins. The domain arrangement is illustrated on the top of the aligned sequences, featuring the NTD (orange), variable acidic tracts (red), and the nuclear localization signal (blue).

**Supplementary Tables:**

**Supplementary Table 1: Sedimentation Velocity – Analytical Ultracentrifugation (SV-AUC) data for *Pf*NPM**

| **Protein Identity** | **Sedimentation**  **Coefficient (S)** | **Theoretical**  **Mol. Mass**  **(kDa)** | **CalculatedMol. Mass**  **(kDa)** | **Frictional**  **Ratio**  **(f/fo)** | **Stokes Radius**  **(nm)** | **RMSD** |
| --- | --- | --- | --- | --- | --- | --- |
| *Pf*NPM NTD | 4.38 | 58.75 | 56.71 | 1.20 | 3.07 | 0.002 |
| *Pf*NPM A1 | 4.40 | 67.50 | 63.90 | 1.35 | 3.56 | 0.005 |
| *Pf*NPM A3 | 4.68 | 102.55 | 98.30 | 1.70 | 5.34 | 0.004 |

**Supplementary Table 2: SAXS data collection and structure parameters for *Pf*NPM**

|  | ***Pf*NPM NTD** | ***Pf*NPM A1** | ***Pf*NPM A3** |
| --- | --- | --- | --- |
| **Data collection parameter** | | | |
| Beamline | BM29 BioSAXS (ESRF) | | |
| Wavelength (Å) | 0.9794 | | |
| Energy (KeV) | 7 – 15 | | |
| Detector | Pilatus3 2M | | |
| Q range (nm^-1^) | 0.025 – 6.0 | | |
| Beam size at sample (µm^2^) | 200 × 200 | | |
| Concentration (mg/ml) | 0.5 – 3.0 | | |
| Sample to detector distance (m) | 2.867 | | |
| Absolute intensity calibration | Water | | |
| Sample volume | 50 µl | | |
| Temperature | 20 °C | | |
| **Structural parameters** | | | |
| *R*g (Å) (from *P(r)*) | 23.44 ± 0.01 | 28.50 ± 0.01 | 43.65 ± 0.06 |
| I(0) (cm^-1^) (from *P(r)*) | 162.4 ± 0.12 | 244.4 ± 0.14 | 369.8 ± 0.38 |
| *R*g (Å) (from Guinier) | 24.00 ± 0.00 | 28.30 ± 0.00 | 42.4 ± 0.01 |
| *I*(0) (cm^-1^) (from Guinier) | 162.4 ± 0.18 | 143.2 ± 0.18 | 366.55 ± 0.46 |
| Dmax (Å) | 69.70 | 86.00 | 145.40 |
| Porod volume (Å^3^) | 97.639 | 140.661 | 203.741 |
| **Molecular mass determination** | | | |
| From Porod volume (*Vp*/1.7) (kDa) | 57.42 | 82.74 | 119.84 |
| Calculated from sequence (kDa) | 11.0 (55) | 12.5 (62.5) | 19.5 (97.5) |
| Chi^2^ (χ^2^) | 4.73 | 26.74 | 33.57 |
| **Modeling parameters** | | | |
| Symmetry | P5 | | |
| DAMAVER (10 DAMMIF) mean NSD | 0.425 ± 0.046 | 0.495 ± 0.057 | 1.244 ± 0.181 |
| **Software employed** | | | |
| Primary data reduction | Beamline Pipeline | | |
| Data processing | PRIMUS | | |
| *ab-initio* analysis | DAMMIF | | |
| Validation and averaging | DAMAVER | | |
| Computation of model scattering | FoXS | | |
| 3D graphic model representation | PyMOL | | |

**Supplementary Table 3: *Pf*NPM Cryo-EM data collection and initial image processing statistics**

| **Data Collection and processing** | |
| --- | --- |
| Magnification | 165,000x |
| Voltage (kV) | 300 |
| Image filter | BioQuantum |
| Slit width (eV) | 20 |
| Electron exposure (e^-^/Å) | 58 |
| Defocus range (μm) | -0.8 to -1.6 |
| Pixel size (Å) | 0.4226 |
| Symmetry imposed | C5 |
| Micrographs | 4679 |
| **Particles picked for 2D classification (no.)** | |
| Template-based particle picking | 1,069,357 |
| Laplacian-of-Gaussian (LoG)-based picking | 1,276,908 |
| Topaz particle-picking | 921,112 |

**Supplementary Table 4: Sedimentation Velocity – Analytical Ultracentrifugation (SV-AUC) data for *Pf*NPM – assembled histone complexes**

| **Protein Identity** | **Sedimentation**  **Coefficient (S)** | **Molecular mass (kDa)** | **Frictional**  **Ratio (f/fo)** | **Stokes Radius (nm)** | **RMSD** |
| --- | --- | --- | --- | --- | --- |
| *Pf*NPM A1 – H2A/H2B | 5.31 | 90.59 | 1.36 | 4.05 | 0.003 |
| *Pf*NPM A3 – H2A/H2B | 5.55 | 123.51 | 1.68 | 5.53 | 0.003 |
| *Pf*NPM A1 – H3/H4 | 5.14 | 120.34 | 1.74 | 5.74 | 0.003 |
| *Pf*NPM A3 – H3/H4 | 6.29 | 144.78 | 1.58 | 5.48 | 0.003 |
| H2A/H2B | 2.08 | 28.32 | 1.49 | 3.04 | 0.002 |
| H3/H4 | 2.64 | 48.20 | 1.69 | 4.11 | 0.003 |

**Supplementary Table 5: SAXS data collection and structure parameters for *Pf*NPM A3 complex with H2A/H2B and H3/H4**

|  | ***Pf*NPM A3 –H2A/H2B** | ***Pf*NPM A3 –H3/H4** |
| --- | --- | --- |
| **Data collection parameter** | | |
| Beamline | BM29 BioSAXS (ESRF) | |
| Wavelength (Å) | 0.9794 | |
| Energy (KeV) | 7 – 15 | |
| Detector | Pilatus3 2M | |
| Q range (nm^-1^) | 0.025 – 6 | |
| Beam size at sample (µm^2^) | 200 × 200 | |
| Concentration (mg/ml) | 0.5 – 3.0 | |
| Sample to detector distance (m) | 2.867 | |
| Absolute intensity calibration | Water | |
| Sample volume | 50 µl | |
| Temperature | 20 °C | |
| **Structural parameters** | | |
| *R*g (Å) (from *P*(r)) | 44.80 ± 0.30 | 48.93 ± 0.20 |
| I(0) (cm^-1^) (from *P*(r)) | 96.69 ± 0.44 | 253.8 ± 0.66 |
| *R*g (Å) (from Guinier) | 44.90 ± 0.13 | 48.93 ± 1.19 |
| *I*(0) (cm^-1^) (from Guinier) | 96.64 ± 0.32 | 253.8 ± 0.64 |
| Dmax (Å) | 139.0 | 158.40 |
| Porod volume (Å^3^) | 229.047 | 283.562 |
| **Molecular mass determination** | | |
| From Porod volume (*Vp/1.7*) (kDa) | 134.733 | 166.80 |
| Calculated from sequence (kDa) | 124.5 | 150.5 |
| Chi^2^ (χ^2^) | 1.38 | 1.93 |
| **Modeling parameters** | | |
| Symmetry | P1 | |
| DAMAVER (10 DAMMIF) mean NSD | 0.862 ± 0.04 | 0.878 ± 0.041 |
| **Software employed** | | |
| Primary data reduction | Beamline Pipeline | |
| Data processing | PRIMUS | |
| *ab-initio* analysis | DAMMIF | |
| Validation and averaging | DAMAVER | |
| Computation of model scattering | FoXSDock | |
| 3D graphic model representation | PyMOL | |

**Supplementary Reference:**

1. Felsenstein, J., *Confidence limits on phylogenies: An approach using the bootstrap.* Evolution, 1985. **39**(4): p. 783-791.
